# Supplementary material for: Exploring and exploiting the rice phytobiome to tackle climate change challenges
Source: Plant Commun. 2024 Sep 3;5(12):101078. doi: 10.1016/j.xplc.2024.101078 (PMC11671768; doi:10.1016/j.xplc.2024.101078)
Supplement: Document S1. Supplemental Tables 1 and 2 and Supplemental Figure 1 [file mmc1.pdf]

Plant Communications, Volume *5*

Supplemental information

Exploring and exploiting the rice phytobiome to tackle climate change challenges

Seyed Mahdi Hosseiniyan Khatibi, Niña Gracel Dimaano, Esteban Veliz, Venkatesan Sundaresan, and Jauhar Ali

Exploring and exploiting the rice phytobiome to tackle climate change challenges

Seyed Mahdi Hosseiniyan Khatibi,[1†] Niña Gracel Dimaano,[1,2†] Esteban Veliz,[3] Venkatesan Sundaresan,[3,4] Jauhar Ali[1]*

Affiliations/institutions

[1]International Rice Research Institute, Los Baños, Laguna, Philippines

[2]College of Agriculture and Food Science, University of the Philippines Los Baños, Laguna, Philippines

[3]College of Biological Sciences, UC Davis

[4]College of Agricultural and Environmental Sciences, UC Davis

***Corresponding author:**

Dr. Jauhar Ali

International Rice Research Institute, Los Baños, Laguna, Philippines

Email: J.Ali@irri.org

† These authors contributed equally and should be considered as co-first authors.

Short Summary:

The rice phytobiome is composed of an intricate network of interdependent and interrelating communities of micro- and macroorganisms and their surrounding environment. Dynamic interactions, facilitated by various communication signals, occur within the rice phytobiome that directly or indirectly influence rice plant growth, development, stress responses to environmental challenges, and overall rice ecosystem functioning. By using novel, data-driven, and systems-level approaches, the rice phytobiome can be accurately reprogrammed to combat climate change threats through the development of prescriptive and predictive analytics for the next-generation precision rice based agricultural systems.

This file includes:

Supplementary Table 1, 2

Supplementary Figure 1

References

Supplementary Table 1. Components of the rice phytobiome.

**Component**	**Composition**	**Examples**	**Key roles/functions**	**References**
Phyllosphere (aboveground) microbiomes				
a. Seed microbiome (outer grain, grain, husk, and outer husk)	Bacteria	*Sphingomonas*, *Acinetobacter*, *Aureimonas, Curtobacterium*, *Enterobacter*, *Exiguobacterium*, *Lactobacillus*, *Microbacterium*, *Pantoea*, *Pseudomonas*, *Methylobacterium fujisawaense*, *M. radiotolerans*, *Bacillus fusiformis*, *Kocuria palustris*, *Pantoea ananatis*, and *Micrococcus luteus,*	Enhance seed quality<br><br>Safeguard young seedlings from diseases<br><br>Increase plant fitness	(Nelson, 2018; Shade et al., 2017)
	Fungi	*Curvularia*, *Moesziomyces*, *Sacrocladium*, *Bipolaris*, *Fusarium equiseti*, *F. oxysporum*, *Epicoccum purpurascens*, *Cladosporium tenuissimum*, *Alternaria alternata*, and *Phoma sorghina*	Endophytic isolates of plant diseases or saprobes	(Fisher and Petrini, 1992)
b. Endosphere microbiome (inside spaces of a plant's stems, roots, leaves, fruits, and seeds)	Beneficial endophytes	*Microbacterium laevaniformans*, *Bacillus tequilensis, Micrococcus* sp., *Pseudomonas*, *Pseudomonas* sp., *Acremonium* sp., *Arthrobotrys* sp., *Fusarium* sp., *Penicillium* sp., *Aspergillus* sp., *Pyricularia* sp.	Positively modulate plant growth, productivity, and stress tolerance<br><br>Secrete secondary metabolites for protection against pathogen attack<br><br>Solubilize phosphate, produce siderophores, reduce sulfate and	(Hardoim et al., 2015; Jana et al., 2022)

			cyanide, and oxidize ammonia<br><br>Enhance water use efficiency<br><br>Induce systemic resistance in plants and stimulate plant growth	
	Commensal endophytes	*Penicillium* sp., *Aspergillus* sp.	Have neutral effects on plant performance	(Hardoim *et al.*, 2015; Rodriguez and Redman, 2008)
c. Plant microbiome (aboveground parts, including leaves, stems, and panicles)	Bacteria	*Proteobacteria*, *Firmicutes*, *Actinobacteria*, *Bacteroidetes*, *Cyanobacteria*, *Acinetobacter*, *Aureimonas*, *Curtobacterium*, *Enterobacter*, *Exiguobacterium*, *Lactobacillus*, *Microbacterium*, *Pantoea*, *Pseudomonas*, *Sphingomonas*	Beneficial, causing positive effects on overall plant growth of rice, or harmful, causing diseases of rice	(Peng et al., 2020)
	Fungi	Ascomycota (Eurotiomycetes, Dothideomycetes, and Sordariomycetes); Basidiomycota (Ustilaginomycetes); Aspergillus		(Peng *et al.*, 2020)
	Viruses	Rice Tungro Bacilliform Virus (RTBV) and Rice Stripe Virus (RSV)	Can cause viral diseases that have detrimental effects on rice productivity	(Deng et al., 2013; Hull, 1996)
	Bacteriophages	*Siphoviridae, Myoviridae*, and *Podoviridae*	Viruses that infect bacteria contribute to shaping the bacterial community structure within the rice phytobiome	(Jain et al., 2023; Zhang et al., 2021)

Rhizosphere and soil (belowground) microbiomes				
a. Rhizosphere microbiome (soil region surrounding the rice roots)	Bacteria	Acidobacteria, Chloroflexi, Firmicutes, and Proteobacteria	Involved in nutrient cycling, plant growth promotion, and disease suppression<br><br>Primarily influenced by factors relating to soil and plants, such as soil type, geographical location, and rice genotype, among others	(Ding et al., 2019; Edwards et al., 2015; Hussain et al., 2018; Santos et al., 2021)
	Fungi	Claroideoglomus, Gigaspora, Redeckera, Retroconis, Schizangiella, Ascomycota, Basidiomycota, and Arbuscular mycorrhiza	Enhance biotic resistance to soil-borne fungal and bacterial pathogens, nematodes, or root-chewing insects<br><br>Improve plant tolerance to abiotic stresses such as shading, high salinity, drought, and heavy metal contamination	(Chang et al., 2021; Wang et al., 2021; Xie et al., 2022)
	Archaea	Methanosaeta, Methanosarcina, Methanobacterium, Methanocella, and Methanoregula	Have a function in methane production	(Conrad et al., 2006; Wang et al., 2018)
	Nematode	Criconemella, Helicotylenchus, Hemicriconemoides, Hirschmanniella, Meloidogyne, and Tylenchorhynchus	Rice root parasites that can cause damage by disrupting the plant's vascular system, leading to decreased nutrient and water uptake<br><br>Predators of other soil-dwelling pests,	(Prot et al., 1994)

			including harmful nematodes and insect larvae<br><br>Certain nematodes have a symbiotic relationship with nitrogen-fixing bacteria	
b.Soil microbiome (soil surrounding a rice plant)	Bacteria	Chloroflexi, Proteobacteria, Acidobacteria, and Actinobacteria	Directly influence plant health and ecosystem functioning<br><br>Crucial for organic matter decomposition, nutrient cycling, and disease suppression	(Hussain et al., 2011; Jiang et al., 2016)
	Fungi	Glomeromycota, Basidiomycota, and Ascomycota		(Jiang et al., 2016; Yuan et al., 2018)
Macrobiome (macroorganims)				
Arthropods	Harmful insect pests	*Chilo suppressalis* (rice stem borer); *Nilaparvata lugens* (brown planthopper); *Nephotettix malayanus* and *N. virescens (green leafhoppers); Leptocorisa oratorius (rice seed bug); Scotinophara spp. (rice black bug); Spodoptera litura (common cutworm); Cnaphalocrocis medinalis (rice leaf folder)*	Damaging insect pests that attack plant parts, thus causing substantial yield losses	(Peng et al., 2016)
	Beneficial insects	*Cheilomenes sexmaculata* (ladybird beetles), *Chrysopa* sp. (lacewing), *Euborellia annulata* (earwigs), *Mantis religiosa* (European mantis), *Diadegma insulare* (parasitic wasp)	Benefit rice by controlling insect pest populations<br><br>Have ecological importance in maintaining rice ecosystem stability	(Peng et al., 2016; Ray and Chakraborty, 2021)
	Spiders	*Atypena formosana* (dwarf spider),	Prey on insect pests that damage the rice	(Ray and

		Oxyopes javanus (lynx spider), *Tetragnatha virescens* and *T. javana, Argiope catenulate*	plant, thus directly affecting the insect pest population	Chakraborty, 2021)
Mollusks and vertebrates	Snails and mollusks	*Pomacea canaliculata* and *P. maculata (golden apple snails)*	Highly invasive and cause damage to rice	(Horgan et al., 2014)
	Rats and birds	*Passer montanus* (Eurasian tree sparrow), *Lonchura malacca* (chesnut munia), *L. punctulata* (scaly-breasted munia), *L. leucogastra* (white-bellied munia)	Can cause yield damage to rice<br><br>Some birds can be beneficial by feeding on rice pests, thus diminishing their population	(Cohen et al., 1998)
Weeds	Grasses, broadleaves, and sedges	*Echinochloa* spp. (barnyard grass); *Cyperus difformis* (small flower umbrella plant); *Cyperus iria* (rice flatsedge); *Leptochloa chinensis* (sprangle top); *Ischaemum rugosum* (Saromacca grass); *Paspalum distichum* (knotgrass); *Fimbristylis littoralis* (fimbristylis); *Ludwigia octovalvis* (water purselane); *Pontederia vaginalis* (pickerel weed); *Sphenoclea zeylanica* (gooseweed)	Affect rice plants via interference through competition with limited resources or growth inhibition due to allelopathy<br><br>Can outcompete rice plants for soil nutrients and water, thus hampering their growth and development<br><br>Can compete for light, leading to shading and reduced photosynthetic activity<br><br>Allelochemicals produced by weed species inhibit rice seed germination and seedling growth	(Rao et al., 2017)

			Various weed species can serve as alternate hosts of many insect pests and diseases	
Human influences				
Chemical inputs	Chemical pesticides and fertilizers	herbicides, insecticides, fungicides, bactericides, molluscicides, chemical nutrient inputs/fertilizers	Have significant influences on plant-microbe interactions, soil health, and rice ecosystem dynamics<br><br>Control the population of insect pests, weeds, and pathogens and alleviate abiotic stresses related to nutrient deficiencies, respectively<br><br>Can alter the diversity and composition of microbial communities, leading to a decrease in beneficial microorganisms such as plant growth-promoting rhizobacteria<br><br>Disruption of the balance between beneficial and harmful organisms, as well as the development of resistance in the target insect pest populations, leading to more significant problems such as pest resurgence and replacement<br>Pesticide residues in rice fields can	(Lo, 2010)

			persist in the soil, thus affecting soil microbial communities, enzymatic activities, and nutrient cycling processes, and can lead to soil contamination and degradation	

Supplementary Table 2. Tasks and predictions in research on the microbiome.

Type of task	Aim of prediction	Suggested approaches	Reference
Analysis of interaction	Interaction of metabolite-microbe	Embedding method	(Brunner et al., 2023)
Analysis of interaction	Co-occurrence patterns of microbe	Embedding method	(Ge et al., 2023)
Analysis of interaction	Plant–pathogen interactions	Regression analysis	(Emmenegger et al., 2023)
Classification of microbial features	Composition of microbiome	Autoencoder method	(Busato et al., 2023)
Classification of microbial features	Profile of metabolic	Autoencoder method	(Li et al., 2023)
Phenotyping task	Disease suppressive soils	CNN method (Convolutional neural networks)	(Khatibi and Ali, 2024; Zhang et al., 2022)
Phenotyping task	Soil microbiome and crop productivity	Random forests	(Chang et al., 2017; Edwards et al., 2018)
Phenotyping task	Cd accumulation features	SVM, RF, LR, KNN, ANN	(Cheng et al., 2023)

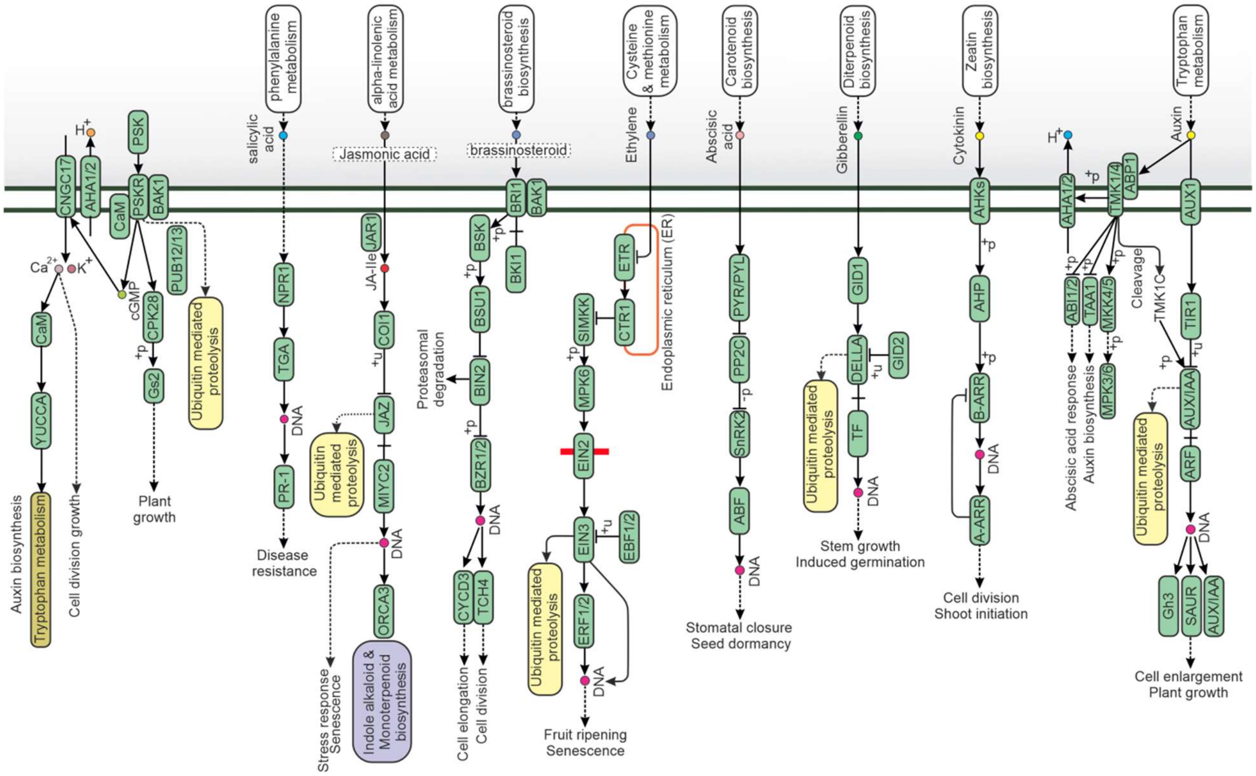

Supplementary Figure 1. The schematic illustration of the general hormones signaling pathways in the rice phytobiome

References

Brunner, J.D., Gallegos-Graves, L.A., and Kroeger, M.E. (2023). Inferring microbial interactions with their environment from genomic and metagenomic data. PLoS Computational Biology **19**:e1011661. https://doi.org/10.1371/journal.pcbi.1011661.

Busato, S., Gordon, M., Chaudhari, M., Jensen, I., Akyol, T., Andersen, S., and Williams, C. (2023). Compositionality, sparsity, spurious heterogeneity, and other data-driven challenges for machine learning algorithms within plant microbiome studies. Current Opinion in Plant Biology **71**:102326. https://doi.org/10.1016/j.pbi.2022.102326.

Chang, H.-X., Haudenshield, J.S., Bowen, C.R., and Hartman, G.L. (2017). Metagenome-wide association study and machine learning prediction of bulk soil microbiome and crop productivity. Frontiers in Microbiology **8**:519. https://doi.org/10.3389/fmicb.2017.00519.

Chang, J., Sun, Y., Tian, L., Ji, L., Luo, S., Nasir, F., Kuramae, E.E., and Tian, C. (2021). The structure of rhizosphere fungal communities of wild and domesticated rice: changes in diversity and co-occurrence patterns. Frontiers in Microbiology **12**:610823. https://doi.org/10.3389/fmicb.2021.610823.

Cheng, Z., Zheng, Q., Shi, J., He, Y., Yang, X., Huang, X., Wu, L., and Xu, J. (2023). Metagenomic and machine learning-aided identification of biomarkers driving distinctive Cd accumulation features in the root-associated microbiome of two rice cultivars. ISME communications **3**:14. https://doi.org/10.1038/s43705-023-00213-z

Cohen, M., Savary, S., Huang, N., Azzam, O., and Datta, S. (1998). Importance of rice pests and challenges to their management. See Ref **30**:145-164.

Conrad, R., Erkel, C., and Liesack, W. (2006). Rice Cluster I methanogens, an important group of Archaea producing greenhouse gas in soil. Current Opinion in Biotechnology **17**:262-267. https://doi.org/10.1016/j.copbio.2006.04.002.

Deng, X., Wang, H., Tang, X., Zhou, J., Chen, H., He, G., Chen, L., and Xu, Z. (2013). Hybrid rice breeding welcomes a new era of molecular crop design. Sci. Sin. Vitae **43**:864-868. https://doi.org/10.1360/052013-299.

Ding, L.-J., Cui, H.-L., Nie, S.-A., Long, X.-E., Duan, G.-L., and Zhu, Y.-G. (2019). Microbiomes inhabiting rice roots and rhizosphere. FEMS microbiology ecology **95**:fiz040. https://doi.org/10.1093/femsec/fiz040.

Edwards, J., Johnson, C., Santos-Medellín, C., Lurie, E., Podishetty, N.K., Bhatnagar, S., Eisen, J.A., and Sundaresan, V. (2015). Structure, variation, and assembly of the root-associated microbiomes of rice. Proceedings of the National Academy of Sciences **112**:E911-E920. https://doi.org/10.1073/pnas.1414592112.

Edwards, J.A., Santos-Medellín, C.M., Liechty, Z.S., Nguyen, B., Lurie, E., Eason, S., Phillips, G., and Sundaresan, V. (2018). Compositional shifts in root-associated bacterial and archaeal microbiota track the plant life cycle in field-grown rice. PLoS biology **16**:e2003862. https://doi.org/10.1371/journal.pbio.2003862.

Emmenegger, B., Massoni, J., Pestalozzi, C.M., Bortfeld-Miller, M., Maier, B.A., and Vorholt, J.A. (2023). Identifying microbiota community patterns important for plant protection using synthetic communities and machine learning. Nature Communications **14**:7983. https://doi.org/10.1038/s41467-023-43793-z.

Fisher, P., and Petrini, O. (1992). Fungal saprobes and pathogens as endophytes of rice (Oryza sativa L.). New Phytologist **120**:137-143. https://doi.org/10.1111/j.1469-8137.1992.tb01066.x.

Ge, J., Li, D., Ding, J., Xiao, X., and Liang, Y. (2023). Microbial coexistence in the rhizosphere and the promotion of plant stress resistance: a review. Environmental Research **222**:115298. https://doi.org/10.1016/j.envres.2023.115298.

Hardoim, P.R., Van Overbeek, L.S., Berg, G., Pirttilä, A.M., Compant, S., Campisano, A., Döring, M., and Sessitsch, A. (2015). The hidden world within plants: ecological and evolutionary considerations for defining functioning of microbial endophytes. Microbiology and molecular biology reviews **79**:293-320. https://doi.org/10.1128/MMBR.00050-14.

Horgan, F.G., Stuart, A.M., and Kudavidanage, E.P. (2014). Impact of invasive apple snails on the functioning and services of natural and managed wetlands. Acta Oecologica **54**:90-100. https://doi.org/10.1016/j.actao.2012.10.002.

Hull, R. (1996). Molecular biology of rice tungro viruses. Annual review of phytopathology **34**:275-297. https://doi.org/10.1146/annurev.phyto.34.1.275.

Hussain, M., Hamid, M.I., Tian, J., Hu, J., Zhang, X., Chen, J., Xiang, M., and Liu, X. (2018). Bacterial community assemblages in the rhizosphere soil, root endosphere and cyst of soybean cyst nematode-suppressive soil challenged with nematodes. FEMS microbiology ecology **94**:fiy142. https://doi.org/10.1093/femsec/fiy142.

Hussain, Q., Liu, Y., Zhang, A., Pan, G., Li, L., Zhang, X., Song, X., Cui, L., and Jin, Z. (2011). Variation of bacterial and fungal community structures in the rhizosphere of hybrid and standard rice cultivars and linkage to CO2 flux. FEMS Microbiology Ecology **78**:116-128. https://doi.org/10.1111/j.1574-6941.2011.01128.x.

Jain, L., Kumar, V., Jain, S.K., Kaushal, P., and Ghosh, P.K. (2023). Isolation of bacteriophages infecting Xanthomonas oryzae pv. oryzae and genomic characterization of novel phage vB_XooS_NR08 for biocontrol of bacterial leaf blight of rice. Frontiers in Microbiology **14**:1084025. https://doi.org/10.3389/fmicb.2023.1084025.

Jana, S.K., Islam, M.M., and Mandal, S. (2022). Endophytic microbiota of rice and their collective impact on host fitness. Current Microbiology **79**:37. https://doi.org/10.1007/s00284-021-02737-w.

Jiang, Y., Liang, Y., Li, C., Wang, F., Sui, Y., Suvannang, N., Zhou, J., and Sun, B. (2016). Crop rotations alter bacterial and fungal diversity in paddy soils across East Asia. Soil Biology and Biochemistry **95**:250-261. https://doi.org/10.1016/j.soilbio.2016.01.007.

Khatibi, S.M.H., and Ali, J. (2024). Harnessing the power of machine learning for crop improvement and sustainable production. Frontiers in Plant Science 15:1417912. https://doi.org/10.3389/fpls.2024.1417912.

Li, J., Gmitter Jr, F.G., Zhang, B., and Wang, Y. (2023). Uncovering Interactions between Plant Metabolism and Plant-Associated Bacteria in Huanglongbing-Affected Citrus Cultivars Using Multiomics Analysis and Machine Learning. Journal of Agricultural and Food Chemistry **71**:16391-16401. https://doi.org/10.1021/acs.jafc.3c04460.

Lo, C.-C. (2010). Effect of pesticides on soil microbial community. Journal of Environmental Science and Health Part B **45**:348-359. https://doi.org/10.1002/2688-8319.12327.

Nelson, E.B. (2018). The seed microbiome: origins, interactions, and impacts. Plant and Soil **422**:7-34. https://doi.org/10.1007/s11104-017-3289-7.

Peng, L., Zhao, Y., Wang, H., Zhang, J., Song, C., Shangguan, X., Zhu, L., and He, G. (2016). Comparative metabolomics of the interaction between rice and the brown planthopper. Metabolomics **12**:1-15. https://doi.org/10.1007/s11306-016-1077-7.

Peng, Y., Tang, J., Hong, M., and Xie, J. (2020). Suppression of rice planthopper populations by the entomopathogenic fungus Metarhizium anisopliae without affecting the rice microbiota. Applied and Environmental Microbiology **86**:e01337-01320. https://doi.org/10.1128/AEM.01337-20.

Prot, J.-C., Soriano, I., and Matias, D. (1994). Major root-parasitic nematodes associated with irrigated rice in the Philippines. Fundamental and Applied Nematology **17**:75-78.

Rao, A., Wani, S., Ahmed, S., Haider Ali, H., and Marambe, B. (2017). An overview of weeds and weed management in rice of South Asia.

Ray, A., and Chakraborty, A. (2021). The edible biota in irrigated, deepwater, and rainfed rice fields of Asia: a neglected treasure for sustainable food system. Environment, Development and Sustainability **23**:17163-17179. https://doi.org/10.1007/s10668-021-01386-0.

Rodriguez, R., and Redman, R. (2008). More than 400 million years of evolution and some plants still can't make it on their own: plant stress tolerance via fungal symbiosis. Journal of experimental botany **59**:1109-1114. https://doi.org/10.1093/jxb/erm342.

Santos, S.S., Rask, K.A., Vestergård, M., Johansen, J.L., Priemé, A., Frøslev, T.G., González, A.M.M., He, H., and Ekelund, F. (2021). Specialized microbiomes facilitate natural rhizosphere microbiome interactions counteracting high salinity stress in plants. Environmental and Experimental Botany **186**:104430. https://doi.org/10.1016/j.envexpbot.2021.104430.

Shade, A., Jacques, M.-A., and Barret, M. (2017). Ecological patterns of seed microbiome diversity, transmission, and assembly. Current opinion in microbiology **37**:15-22. https://doi.org/10.1016/j.mib.2017.03.010.

Wang, C., Jin, Y., Ji, C., Zhang, N., Song, M., Kong, D., Liu, S., Zhang, X., Liu, X., and Zou, J. (2018). An additive effect of elevated atmospheric CO2 and rising temperature on methane emissions related to methanogenic community in rice paddies. Agriculture, Ecosystems & Environment **257**:165-174. https://doi.org/10.1016/j.agee.2018.02.003.

Wang, Y., Bao, X., and Li, S. (2021). Effects of arbuscular mycorrhizal fungi on rice growth under different flooding and shading regimes. Frontiers in Microbiology **12**:756752. https://doi.org/10.3389/fmicb.2021.756752.

Xie, H., Chen, Z., Feng, X., Wang, M., Luo, Y., Wang, Y., and Xu, P. (2022). L-theanine exuded from Camellia sinensis roots regulates element cycling in soil by shaping the rhizosphere microbiome

assembly. Science of The Total Environment **837**:155801. https://doi.org/10.1016/j.scitotenv.2022.155801.

Yuan, J., Zhao, J., Wen, T., Zhao, M., Li, R., Goossens, P., Huang, Q., Bai, Y., Vivanco, J.M., and Kowalchuk, G.A. (2018). Root exudates drive the soil-borne legacy of aboveground pathogen infection. Microbiome **6**:1-12. https://doi.org/10.1186/s40168-018-0537-x.

Zhang, J., Cook, J., Nearing, J.T., Zhang, J., Raudonis, R., Glick, B.R., Langille, M.G., and Cheng, Z. (2021). Harnessing the plant microbiome to promote the growth of agricultural crops. Microbiological Research **245**:126690. https://doi.org/10.1016/j.micres.2020.126690.

Zhang, Z., Zhang, Q., Cui, H., Li, Y., Xu, N., Lu, T., Chen, J., Penuelas, J., Hu, B., and Qian, H. (2022). Composition identification and functional verification of bacterial community in disease-suppressive soils by machine learning. Environmental Microbiology **24**:3405-3419. https://doi.org/10.1111/1462-2920.15902.
