# Supplementary material for: Exploring and exploiting the rice phytobiome to tackle climate change challenges
Source: Plant Commun. 2024 Sep 3;5(12):101078. doi: 10.1016/j.xplc.2024.101078 (PMC11671768; doi:10.1016/j.xplc.2024.101078)
Supplement: Document S2. Article plus supplemental information [file mmc2.pdf]

Plant Communications
Review article

CellPress
Partner Journal

Exploring and exploiting the rice phytobiome to tackle climate change challenges

Seyed Mahdi Hosseiniyan Khatibi[1,5], Niña Gracel Dimaano[1,2,5], Esteban Veliz[3], Venkatesan Sundaresan[3,4] and Jauhar Ali[1,*]

[1]International Rice Research Institute, Los Baños, Laguna, Philippines

[2]College of Agriculture and Food Science, University of the Philippines Los Baños, Los Baños, Laguna, Philippines

[3]College of Biological Sciences, University of California, Davis, Davis, CA, USA

[4]College of Agricultural and Environmental Sciences, University of California, Davis, Davis, CA, USA

[5]These authors contributed equally to this article

***Correspondence: Jauhar Ali (j.ali@irri.org)**

https://doi.org/10.1016/j.xplc.2024.101078

ABSTRACT

The future of agriculture is uncertain under the current climate change scenario. Climate change directly and indirectly affects the biotic and abiotic elements that control agroecosystems, jeopardizing the safety of the world's food supply. A new area that focuses on characterizing the phytobiome is emerging. The phytobiome comprises plants and their immediate surroundings, involving numerous interdependent microscopic and macroscopic organisms that affect the health and productivity of plants. Phytobiome studies primarily focus on the microbial communities associated with plants, which are referred to as the plant microbiome. The development of high-throughput sequencing technologies over the past 10 years has dramatically advanced our understanding of the structure, functionality, and dynamics of the phytobiome; however, comprehensive methods for using this knowledge are lacking, particularly for major crops such as rice. Considering the impact of rice production on world food security, gaining fresh perspectives on the interdependent and interrelated components of the rice phytobiome could enhance rice production and crop health, sustain rice ecosystem function, and combat the effects of climate change. Our review reconceptualizes the complex dynamics of the microscopic and macroscopic components in the rice phytobiome as influenced by human interventions and changing environmental conditions driven by climate change. We also discuss interdisciplinary and systematic approaches to decipher and reprogram the sophisticated interactions in the rice phytobiome using novel strategies and cutting-edge technology. Merging the gigantic datasets and complex information on the rice phytobiome and their application in the context of regenerative agriculture could lead to sustainable rice farming practices that are resilient to the impacts of climate change.

Key words: artificial intelligence, climate change, rice microbiome, rice phytobiome, microbial ecology, rhizosphere engineering

Hosseiniyan Khatibi S.M., Dimaano N.G., Veliz E., Sundaresan V., and Ali J. (2024). Exploring and exploiting the rice phytobiome to tackle climate change challenges. Plant Comm. **5**, 101078.

INTRODUCTION

The world's population is predicted to reach 9.7 billion by 2050 (UN, 2017). To meet the demand for food, crop productivity should rise by 60%–100% (Hunter et al., 2017). Climate change and catastrophes linked to it directly affect the biotic and abiotic elements that control agroecosystems by altering temperatures and precipitation patterns, while indirectly influencing pest dynamics, soil quality, and water resource management. These changes collectively impact the microbial communities, plant health, and overall productivity of agroecosystems, thus jeopardizing the safety of the world's food supply (Challinor et al., 2014; Dresselhaus and Hückelhoven, 2018; CHANGE, 2019; Raza et al., 2019). Meanwhile, more than 50% of the world's population consumes rice (Ding et al., 2014). Rice is grown in regions that already experience several climatic extremes and are

Published by the Plant Communications Shanghai Editorial Office in association with Cell Press, an imprint of Elsevier Inc., on behalf of CSPB and CEMPS, CAS.

This is an open access article under the CC BY license (http://creativecommons.org/licenses/by/4.0/).

susceptible to them (Zhu and Troy, 2018; Vogel et al., 2019). Any interruption to rice as an essential food source could have serious consequences. One novel approach to mitigating the costs of climate change in the rice system is understanding and exploiting the rice phytobiome and its interrelated components that could have crucial roles in coping with the impacts and hazards of climate change.

The term phytobiome refers to the entire system of plants, the environment in which they grow, and all the species that live there (Leach et al., 2017). Dynamic phytobiome interactions between biotic and abiotic elements maintain agroecosystems and natural ecosystems. Through various processes, including nutrient recycling, competition for resources, antagonism, and chemical signals, the phytobiome complex interacts with other members of its network, including microorganisms, the environment, and host plants, to preserve ecological harmony in the phytobiome. In addition to maintaining natural ecosystems, the phytobiome can contribute to the development of crops with increased yield, disease resistance, and ecological adaptability (Leach et al., 2017; Trivedi et al., 2020).

The main focus of phytobiome studies, among the biotic variables, is the plant microbiome, which refers to the microbial populations connected to plants (Trivedi et al., 2020; Chouhan et al., 2021a). As in the human body, microorganisms inhabit the majority of plant components, including the root, leaf, stem, and flower, and they interact to influence the health of the plant (Chouhan et al., 2021b). The direct and crucial effects of the plant microbiome on plant production and health have been brought to light by recent studies (Trivedi et al., 2020; Chouhan et al., 2021a). This has given rise to proposals for different methods of using plant microbiomes to improve sustainable farming techniques by boosting nutrient availability and nutrient use efficiency, as well as boosting defense against diseases and pests (Singh et al., 2018; Verma, 2018). The species of plants, their tissues and developmental phases, and the environments in which they grow affect the makeup of plant microbiomes (Fitzpatrick et al., 2020) and vice versa. Microbes can benefit host plants via modulation of biochemical and molecular signals; mineralization of organic matter; improvement of nutrient acquisition and uptake; P-solubilization; Fe-chelation of heavy metals and organic pollutants; pathogen defense and antagonism; induction and stimulation of resistance to diseases and abiotic stresses; enhancement of drought tolerance; and improvement of rice physiological properties, biomass production, and grain yield (Palmer et al., 2023). However, some microbes, particularly pathogenic ones, can have detrimental effects such as irreversible damage to and diseases in host plants. Others such as rhizobia, mycorrhizal fungi, and actinomycetes can decompose root nodules, thus diminishing nutrient uptake and mobilization efficiency and leaching plant nutrients. All the components of the phytobiome have a microbiome, which includes their co-evolving interactions, and there is a continuous link among them (Girard et al., 2023).

High-throughput sequencing (HTS), also known as next-generation sequencing, and omics approaches such as metagenomics, metaproteomics, metabolomics, and metatranscriptomics have facilitated analyses of the functions of microbial communities and microbial diversity in sophisticated environmental samples. HTS-based research can also be performed on microbial communities associated with plants to fully understand their involvement in supporting the health and fitness of their host plants. Recently, scientists have been concentrating on the newest frontier of metagenomics to develop a comprehensive database of phytobiomes alongside other omics technologies to better understand interactions between the plant microbiome and its environment, as well as within the plant system. To boost our understanding of and research on the dynamics, signaling, and interactions of the rice phytobiome and decipher this super-high complexity, an interdisciplinary and systematic approach to deciphering the rice phytobiome is needed. This can be accomplished using novel approaches such as machine learning (ML), deep learning (DL), and genome editing (Zhan et al., 2022). Our review decodes the complex interactions within the rice phytobiome, offering insights into their practical significance as well as highlighting novel cutting-edge technological approaches with future perspectives to harness their potential applications for rice improvement and food security in the face of climate change.

RICE PHYTOBIOME NETWORK

The rice phytobiome is formed by an intricate network of interdependent and interrelating communities of micro- and macroorganisms and their environment. A rice plant co-exists and dynamically interacts with different groups of organisms, each with its own organizations, functions, and levels of complexity, and each displaying a range of lifestyles, such as mutualistic, pathogenic, and commensal relationships. Each member of the phytobiome interacts with the rice plant, relying on it as a host and source of resources necessary for survival. In turn, members can benefit or negatively affect the rice plant by aiding its growth and stress tolerance or competing with it for available resources. Neutral interactions occur when the presence of certain phytobiome members does not have a noticeable impact on the rice plant. These complex interactions are dynamic and are greatly influenced by climatic, edaphic, and other environmental factors. Figure 1 offers a comprehensive picture of the complex biotic components, communication signals, and intricate interactions in the rice phytobiome system, as well as all evident climatic and edaphic factors that influence the overall health, fitness, and stress resilience of the rice plant and the sustainability of the rice ecosystem. A comprehensive understanding of interactions and signaling in the rice phytobiome could lead to innovative strategies that could enhance rice crop health and help sustain rice ecosystem production, including helping to combat the effects of climate change.

MICROBIOME AND MACROBIOME COMPONENTS

A vital component of the rice phytobiome is the plant microbiome (Trivedi et al., 2020), which refers to the diverse microbial communities that colonize the rice phyllosphere (the seeds, endosphere, and aboveground plant parts) (Sohrabi et al., 2023) and rhizosphere (the soil region surrounding the rice roots) (Fitzpatrick et al., 2020). The microbial communities of each rice compartment are outlined, with examples of taxa and

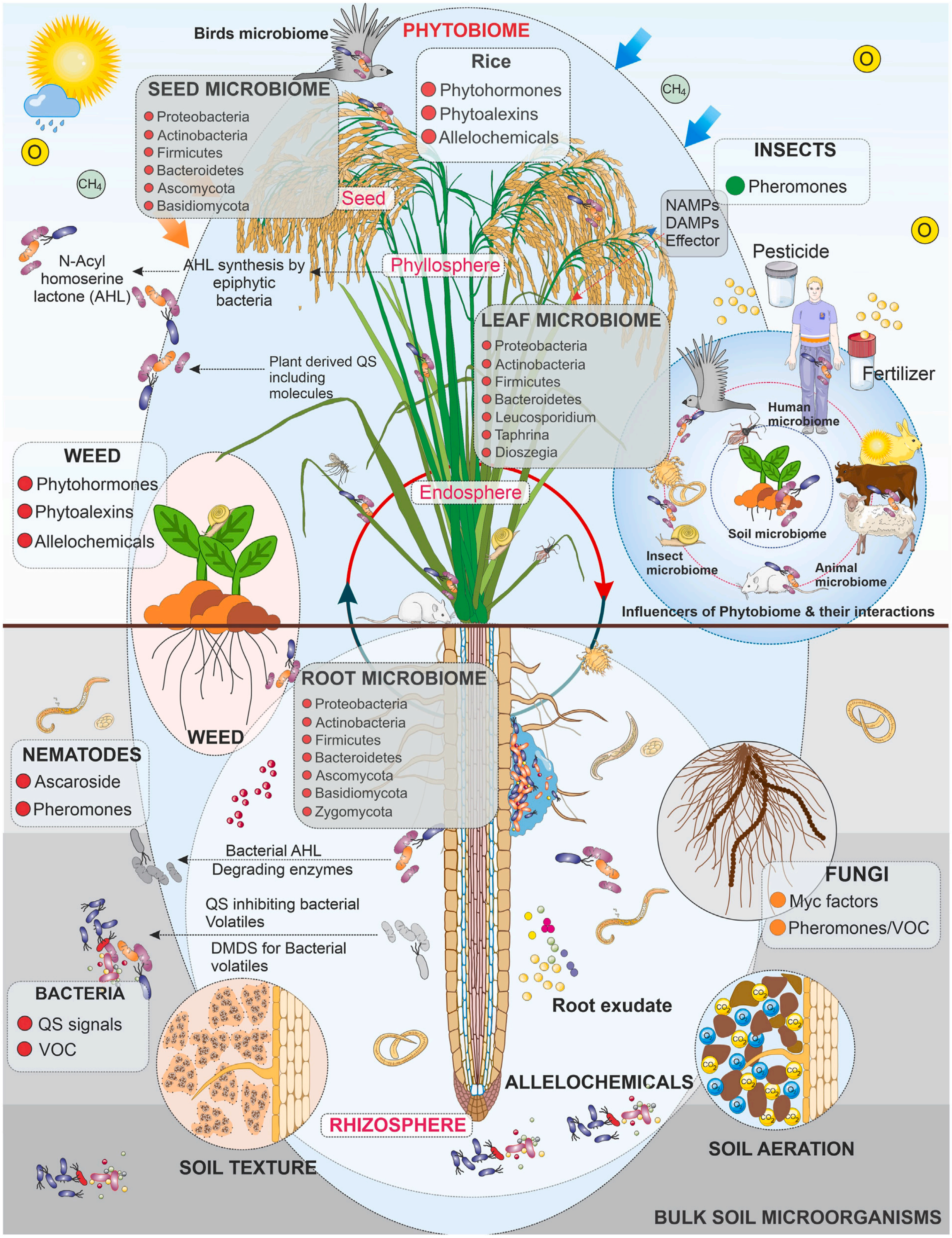

Figure 1. Complex interactions in the rice phytobiome network.

The rice phytobiome network is composed of a community of microorganisms (bacteria, fungi, and viruses) that colonize rice plant compartments (seed, phyllosphere, endosphere, rhizosphere) and soil and macroorganisms (arthropods, weeds, rodents, birds, vertebrates, and other phytobiome influencers); these organisms interact via communication signals (e.g., phytohormones, phytoalexins, allelochemicals, pheromones, QS autoinducers, VOCs, Myc factors, AHL) and are influenced by climatic and edaphic factors (e.g., atmospheric gases, soil texture, soil aeration) and management practices (e.g., application of chemical pesticides and fertilizer, cultural practices).

some of their functions, in Supplemental Table 1. The ecological mechanisms behind the formation of microbiomes in various plant compartments are poorly understood. Recent data have revealed that some plant microbiome taxa, referred to as the core microbiome, are present in samples of most plant species, regardless of geographic, climatic, or management factors (Hamonts et al., 2018). It is hypothesized that although seed transmission of microbial taxa can occur, most taxa are derived from the surrounding bulk soil. Plant genotypes, environmental factors, and management techniques effectively filter the bulk soil microbiome, leaving a subset of plant-associated microorganisms that constitute the plant microbiome (Edwards et al., 2015). The network of the plant microbiome is intricate and interwoven. Hub taxa, which refer to the microbes that are identified as nodes in network analyses and thus correlated to a large number of other taxa, could be a primary target for *in situ* control of the plant microbiome to enhance sustainable production, because any changes in this area can substantially affect both the core and whole-plant microbiome (Singh et al., 2018). Hence, recognizing the composition and dynamics of the rice microbiome across rice compartments is essential for improving rice productivity and disease management and developing climate-resilient rice in sustainable rice systems. Aside from the diverse microorganisms that coexist in the rice phytobiome are found the more prominent and visible organisms, termed macroorganisms, including beneficial and harmful insects, arthropods, mollusks, earthworms, snails, rodents, birds, and other vertebrates that affect rice production, biodiversity, and ecosystem functioning. Figure 1 and Supplemental Table 1 display an overview of phytobiome components at the macro and micro levels.

RICE PHYTOBIOME SIGNALING AND INTERACTIONS

Dynamic interactions facilitated by various communication signals occur within the rice phytobiome that directly or indirectly influence rice plant growth, development, and stress response to environmental challenges, as well as overall rice ecosystem functioning. In this section, we discuss the intricate signaling and interactions between rice and all biotic components that shape the composition and function of the rice phytobiome and can be manipulated to enhance rice plant health and productivity and improve rice resilience to climate change.

Phytohormone and secondary metabolite signaling in the rice phytobiome

Phytohormones and secondary metabolites are the communication signals that plants produce to interact with other organisms and their environment and to respond to various stimuli (Supplemental Figure 1). The synthesized phytohormones and secondary metabolites are released into the surrounding environment via volatilization, foliar leaching, root exudation, decomposition of plant residue, and debris incorporation into soils (Chou, 1990).

Plants have developed a network of signaling events that activate defense responses by producing defensive compounds. Microorganisms are perceived by plants when microbe-associated molecular patterns (MAMPs) are detected by pattern recognition receptors (PRRs) on the host-plant surface, thus activating MAMP-triggered immunity. PRRs also perceive damage-associated molecular patterns that are produced post-infection to initiate defense mechanisms (Boller and Felix, 2009). In rice, the key regulators of plant response to pathogen attacks and microbial colonization are the plant hormones salicylic acid (SA), jasmonic acid (JA), abscisic acid (ABA), and ethylene (ET) (Pieterse and Van Loon, 1999; Avanci et al., 2010; Yamada et al., 2012). JA and ET regulate responses to necrotrophic pathogens, whereas SA mediates defense against biotrophic pathogens (Glazebrook, 2005). JA signaling plays an important role in the defense responses of rice against bacterial blight caused by *Xanthomonas oryzae* pv. *oryzae* (Yamada et al., 2012). OsWRKY13, an activator of the SA-dependent pathway and a suppressor of JA-dependent pathways, mediates rice resistance to bacterial blight and fungal blast (Qiu et al., 2007).

Phytoalexins are plant-produced inducible secondary metabolites that possess antimicrobial activity toward phytopathogens (Ahuja et al., 2012; Großkinsky et al., 2012). Diterpenoid phytoalexins are implicated in defense against parasitic nematodes and alteration of nematode communities in the rice rhizosphere (Desmedt et al., 2022). The diterpenoids momilactone A and B were identified as phytoalexins in rice leaves infected with *Magnaporthe oryzae* (blast fungus) (Cartwright et al., 1981). Other diterpenoid phytoalexins (e.g., phytocassanes, oryzalexins) have been identified in rice with pathogen infections (Akatsuka et al., 1985; Kato et al., 1993; Koga et al., 1995). The flavonoid sakuranetin, a key phytoalexin in rice that shows strong antimicrobial activity, accumulated to high levels in leaves in response to blast infection (Kodama et al., 1992). Phytoalexins and oxylipins also govern host–fungal pathogen interactions by acting as signals that modulate sporogenesis and mycotoxin biosynthesis (Gao and Kolomiets, 2009).

Volatile organic compounds (VOCs) emitted by plants have been shown to attract beneficial microbes, such as plant growth-promoting rhizobacteria (PGPR), and induce systemic resistance against pathogens (Liu and Brettell, 2019). Moreover, plants that mimic fungal VOCs can attract beneficial insects such as pollinators (Kaiser, 2006). Other plant volatiles suppress pheromone signaling by decreasing the responsiveness of insect olfactory neurons (Hatano et al., 2015).

Root exudates are composed of amino acids, organic acids, sugars, and secondary metabolites that are secreted by plants into the rhizosphere through diffusion, ion channel pumping, and vesicle transport (Lyu and Smith, 2022). Rice root exudates affect the chemical and physiological characteristics of the soil, the microbial community, and the growth of other competitive plant species in the rice phytobiome. Numerous root exudates transmit rhizosphere signals (e.g., strigolactones, coumarins, flavonoids), thus influencing nearby plants and rhizosphere microbial populations (Badri and Vivanco, 2009; Asaf et al., 2020). In addition, root exudates facilitate the physicochemical adaptation of plants to the soil environment, offering nutrients for the early colonization of soil microbial populations. Therefore, root exudates aid plant biological adaptability in soil environments by enlisting bacteria that improve the rhizosphere.

Allelochemicals are specific types of secondary metabolites released by plants that influence the growth, survival, and reproduction of other organisms (Mizutani, 1999). Allelopathic rice varieties release allelochemicals that may exert inhibitory or stimulatory effects on germinating weed seeds, affect the activity of microbial and pathogenic diversity in the rice phytobiome, and modify soil characteristics (Amb and Ahluwalia, 2016). Allelochemicals produced by rice are highly influenced by the interaction of rice with specific microorganisms, such as myxobacteria in the rhizosphere (Qu and Wang, 2008; Lin, 2013). Allelochemicals can also manipulate microbial ecology by influencing rhizosphere microbes and plant pathogens (Einhellig, 1995). In addition, the soil microbiome can directly or indirectly affect allelopathic interactions between rice and weeds, as the activity of soil-dwelling microorganisms can speed up the conversion and modification of allelochemicals. These processes can alter the chemical and biological characteristics of the allelochemicals, possibly making them more effective, dormant, or even detrimental to other organisms (Schandry and Becker, 2020). Putative allelochemicals found in rice include phenolic acid compounds (*p*-coumaric acid, *p*-hydroxybenzoic acid, ferulic acid, and vanillic acid) (Rimando et al., 2001; Seal et al., 2004). Phenolic acids (e.g., *p*-coumaric acid, ferulic acid, *p*-hydroxybenzoic acid, and oxalic acid) are rice allelochemicals produced during residue decomposition, potentially fixed by humic acid or soil aggregate structural components, and deposited in the rhizosphere to prevent rice and weed seedling growth (Rimando et al., 2001; Seal et al., 2004). Other allelochemicals, such as terpenoids and flavonoids, can suppress weeds associated with rice, such as *Echinochloa* spp. The terpenoid momilactone B was found to have a highly inhibitory effect on target weeds at a low dosage (Kato-Noguchi, 2004; Kato-Noguchi and Ino, 2005; Wang et al., 2010; He et al., 2012). Caffeic acid, *trans*-ferulic acid, and *p*-hydroxybenzoic acid were found in the exudates of allelopathic rice cultivars (Seal et al., 2004). Fatty acid esters, unsaturated ketones, and polycyclic aromatic compounds were also identified as allelochemicals, and some alkaloids from the ethyl acetate fraction of rice extracts were found to be phytotoxic to *Echinochloa crus-galli* (barnyardgrass) (Kim and Shin, 2008).

Microbial signaling in the rice phytobiome

Microbes in the rice phytobiome engage in intercellular communication through the secretion of signaling molecules. Quorum sensing (QS) autoinducers such as *N*-acyl homoserine lactones (AHLs), lipid-like diffusible signal factors, and signaling peptides are produced by both beneficial and pathogenic microbes, influencing their colonization, virulence, and interactions with the rice host (Poonguzhall et al., 2007; Chung et al., 2011; Leach et al., 2017; Viswanath et al., 2020). Degradation of plant cell walls necessary for plant pathogenicity and colonization activities such as biofilm formation, adhesion and motility, pathogenicity, and the production of enzymes that degrade cell walls are all mediated by autoinducers. There has been a thorough analysis of the bacterial signaling mechanisms by which lipo-chitooligosaccharides (LCOs; Nod factors) and exopolysaccharides help to establish the nodule symbiosis and confer host specificity. Some homoserine lactones can be actively absorbed by plant roots, perceived by them, or transported throughout the plant, with quantifiable impacts on hormones and development (Hartmann et al., 2014). ABA produced by fungal pathogens activates pathogenesis and enhances plant susceptibility (Spence et al., 2015). VOCs produced by microbes have antifungal properties and are inhibitory to pathogen growth. *Pseudomonas* sp., *Enterobacter* sp., *Ralstonia* sp., *Bacillus* sp., *Arthrobacter* sp., *Brevibacillus* sp., and *Paenisporosarcina* sp. showed various abilities to inhibit *Rhizoctonia solani* growth via VOCs (Wang et al., 2021). VOCs released by bacteria and fungi also influence insect behavior (Davis et al., 2013). Synthesis of the volatile chemical indole can impact virulence factors, stress survival mechanisms, and biofilm formation of the surrounding bacteria (Lee et al., 2015). Natural selection may have favored indole signaling as a ubiquitous physiological code owing to the convergent development of indoleacetic acid production in bacteria, microalgae, fungi, and plants (Fu et al., 2015). Cross-kingdom signaling between rice and its associated microbes is crucial for establishing mutualistic associations and defense responses. For instance, the LCO plant–microbe symbiosis factors, produced by rhizobia (as Nod factor) and mycorrhizal fungi (as Myc factor), induce nodulation in legumes and mycorrhization in rice and other plants, respectively (Gough and Cullimore, 2011; Sun et al., 2015; Barker et al., 2017). Extracellular vesicles secreted by rice roots and microbes have also been implicated in intercellular communication, facilitating the exchange of signaling molecules and genetic material (Roth et al., 2019; Stotz et al., 2022).

Rice–insect signaling and interaction

The success of insect herbivores and plant disease vectors depends on pheromone communication. Through volatile pheromones or semiochemicals, insects and other arthropods communicate with one another about danger, social standing, food availability, and mating. Plant–insect interactions are influenced by release of the insect's oral secretions (saliva, gut regurgitant) and oviposition fluids into the plant. The successful feeding of insects depends on saliva properties and functions, and compounds in insect saliva can elicit or inhibit plant immune responses to insect attacks (Miles, 1999). Plants perceive various insect herbivores by integrating diverse environmental cues (e.g., insect mechanostimulation on plant surfaces and contact with salivary components). Upon perception, regulatory responses, including multiple phytohormones, are triggered, with the JA pathway controlling host resistance. Furthermore, the interaction of numerous hormone response pathways translates initial perception into optimized responses to enhance plant fitness under herbivore attack (Erb et al., 2012). Proteins encoded for insect resistance in rice perceive the insect effectors and activate defense pathways such as expression of defense-related genes, mitogen-activated protein kinases, transcription factors, and plant hormones, as well as defense mechanisms against insects, such as trypsin proteinase inhibitors, callose deposition, green leaf volatiles, and secondary metabolites (Du et al., 2020). Elicitors identified in insect oral secretions include β-glucosidase, fatty acid–amino acid conjugates, volicitin, and caeliferins, which activate the JA signaling pathway that controls defense responses against insects (Mattiacci et al., 1995; Alborn et al., 2007; Aggarwal et al., 2014). In response to injury and herbivory, plants produce

JA and the amino acid conjugate jasmonoyl-l-isoleucine (JA-Ile) as key defense signals (Erb et al., 2012; Fukumoto et al., 2013). JA and ET regulate responses to chewing insects and herbivores, whereas SA mediates defense against phloem-feeding insects (Glazebrook, 2005). Interactions between insects and viral pathogens are significant for the transmission of economically damaging rice diseases. Insect pests such as planthoppers and leafhoppers can be vectors of viral diseases such as tungro and hopperburn, thus causing indirect damage to rice (Fujita et al., 2013). At the same time, beneficial bacteria can aid in the rice plant's defense against insect pests. For instance, bacteria in the honeydew of brown planthopper (BPH) activate rice defense responses, such as the release of volatile compounds and the accumulation of phytoalexins to attract natural enemies of BPH (Wari et al., 2019).

THE RICE PHYTOBIOME ECOSYSTEM UNDER CLIMATE-CHANGE CHALLENGES

Climate-change factors that trigger increasing occurrences of abiotic and biotic stress affect the structure and function of the rice phytobiome. Under various stresses, plants undergo stress perception, signal processing, and stress response optimization, thus maximizing resistance while limiting costs and side effects (Ravanbakhsh et al., 2018). Figure 2 provides a diagram of rice phytobiome signaling and optimization of stress response under the adverse effects of climate change. Intricate signaling pathways facilitate the activation of defense mechanisms and adaptive responses. They are enhanced by microbiome communities, thus enabling rice plants to have better fitness and improved health, mitigate the detrimental consequences of climate-induced stressors, and achieve a sustainable rice ecosystem. Notable examples reported in rice on the functions of these signaling pathways with the modulation of some macro-/microbial populations found in the rice phytobiome under climatic stresses are discussed below.

Heat stress

In response to heat stress due to elevated temperatures, plants exhibit a variety of defense mechanisms, such as the activation of hormone-signaling pathways and heat shock proteins that increase their thermotolerance. ET-mediated signaling is involved in the reduction of oxidative damage, maintenance of chlorophyll content, and improvement of thermotolerance in rice seedlings under heat stress (Wu and Yang, 2019). Also, in rice seedlings, free radical H_2O_2 and NO were found to act as signal molecules and increase salt and heat tolerance (Uchida et al., 2002). Changing temperature regimes influence the rhizosphere microbiota, thus affecting rice growth and heat-stress tolerance. Microbes contribute to the heat-stress tolerance of rice by detoxifying chemicals or releasing protective substances to withstand desiccation (Horvath, 1972; Ruíz-Sánchez et al., 2011). Meanwhile, the effect of high temperatures on the microbial composition of the rhizosphere alters nutrient availability and plant–microbe interactions (Haugwitz et al., 2014). As shown in several studies, rising temperatures and elevated atmospheric CO_2 can modify the rhizosphere or root and soil microbial community (Yue et al., 2007; Peng et al., 2008; Das and Adhya, 2012; Lu et al., 2015; Peltoniemi et al., 2016), but they have no significant effects on the composition and abundance of methanogenic communities (Angel et al., 2012; Liu et al., 2012, 2016).

Drought

Drought results in a major restructuring of the rice root microbiome, marked by enrichment of Actinobacteria and *Chloroflexi* (Santos-Medellín et al., 2017). After prolonged drought, the endosphere community shows delayed recovery, including persistence of taxa such as *Streptomyces* that are capable of promoting root growth, possibly conferring tolerance to future droughts (Santos-Medellin et al., 2021). Rhizosphere microbes can boost plant drought tolerance (Welbaum et al., 2004; Mendes et al., 2011; Berendsen et al., 2018). Recent studies have highlighted the roles of specific microbial taxa, often found in the phyla Actinobacteria, Proteobacteria, and Firmicutes, in enhancing drought tolerance by modulating phytohormone production, nutrient availability, and stress-responsive gene expression (Berendsen et al., 2018). Under drought, some root exudates can act as signaling molecules, altering plant–microbe, microbe–microbe, and plant–plant interactions. Root exudates also serve as nutrients for microbes and can alter the physical and chemical properties of the soil. Several studies have shown that the amount and composition of root exudates change under drought conditions (Song et al., 2012; Karst et al., 2017; Canarini et al., 2019, 2021). We can observe that, as the severity of a drought increases, the total volume of root exudates declines while the ratio of carbon allocation to root exudates rises. Alterations in root exudate composition and soil moisture significantly influence the structure and function of the rice rhizosphere microbiota under drought stress. For instance, phytosiderophore secretion decreases in drought-stressed environments, directly or indirectly promoting the growth of Actinobacteria genera such as *Streptomyces,* which can promote plant growth (Omae and Tsuda, 2022). Santos-Medellín et al. (2017) also observed changes in the fungal community of the rice rhizosphere and root endosphere under drought conditions, although the effects were smaller than those observed for the bacterial community.

Salinity and alkalinity

High soil salinity decreases microbial diversity and changes the community structure of rhizosphere microbes, with a decrease in beneficial bacteria and an increase in halotolerant and halophilic organisms, thus leading to imbalanced nutrient cycling and decreased plant health (Abdul Rahman et al., 2021). Crop plants subsequently inoculated with PGPR have significantly improved salt tolerance and plant growth under saline soil conditions (Wang et al., 2022; Phour and Sindhu, 2023). The rhizosphere microbiome significantly enhances salinity tolerance through various mechanisms, including the modulation of phytohormone biosynthesis and signaling pathways (e.g., indole acetic acid, gibberellic acid, brassinosteroids, ABA, JA); accumulation of osmoprotectants (e.g., proline, glycine betaine, sugar alcohols); production of compounds such as antioxidants, 1-aminocyclopropane-1-carboxylate (ACC) deaminase enzymes, exopolysaccharides, organic acids, osmoprotectants, nitric oxide, and siderophores; regulation of ion transporters; and mediation by PRRs that sense

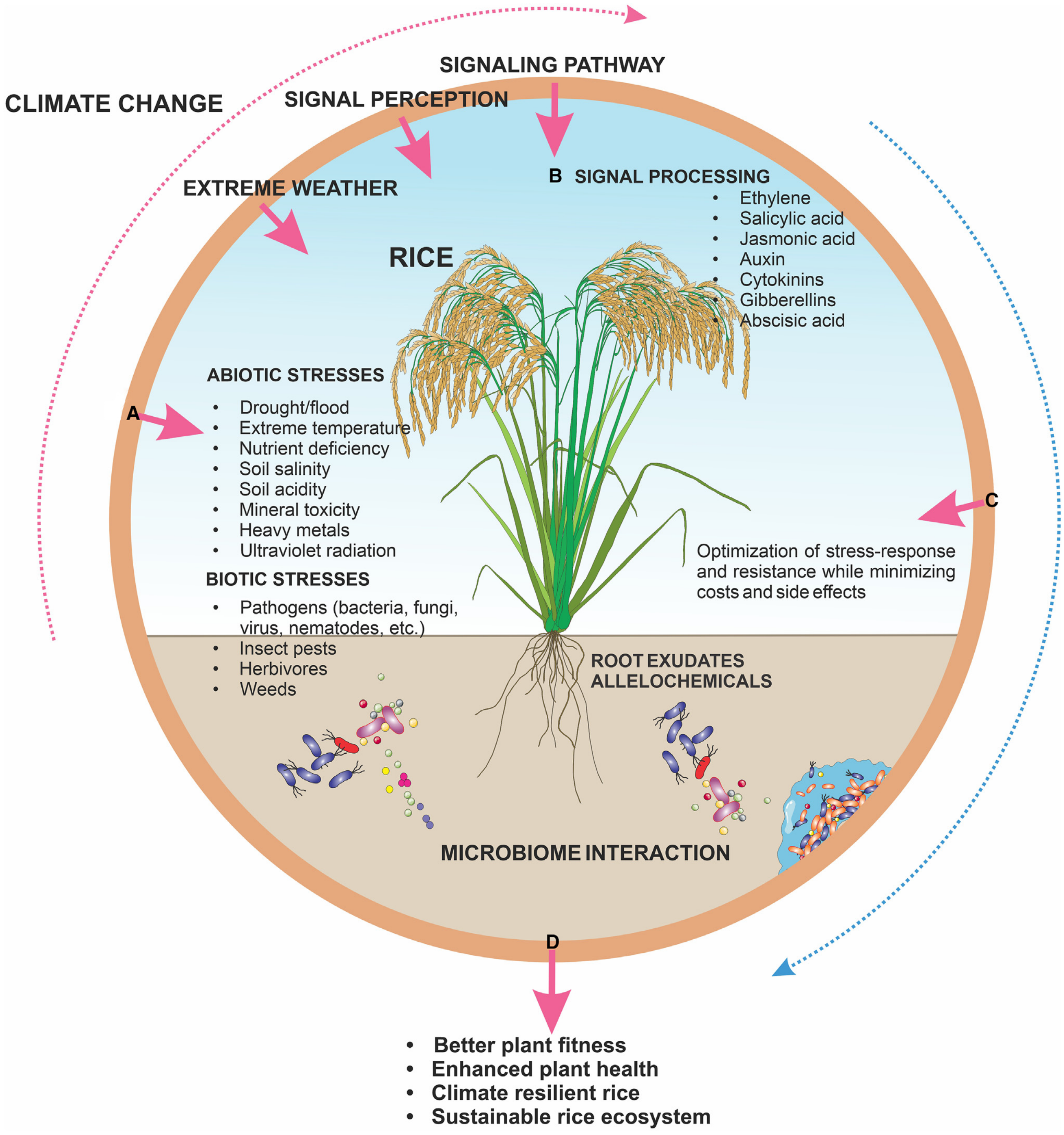

Figure 2. Manipulatable components of the rice phytobiome for optimized stress response to climate change .
Rice phytobiome signaling networks and optimization of stress response under **(A)** biotic and abiotic stresses triggered by climate change. The intricate **(B)** signaling pathways facilitate the **(C)** activation and optimization of defense mechanisms and adaptive responses. They are enhanced by microbiome communities, enabling rice plants to have **(D)** better plant fitness and improved plant health, mitigate the detrimental effects of climate-induced stressors, and achieve a sustainable rice ecosystem.

microbe- or plant-derived molecules (Saijo and Loo, 2020). However, plant tolerance to salinity can be negatively regulated by SA signaling through crosstalk with ABA signaling (Berens et al., 2019). Similar to salinity stress, alkalinity stress alters the composition of the rice root-associated microbial community, leading to impaired nutrient uptake and decreased plant growth. Some genera of rhizosphere alkaliphilic bacteria in the Bacillaceae family (e.g., *Alkalibacillus*, *Bacillus*, *Haloalkalibacillus*) tolerate alkalinity stress through the cytoplasmic membrane proton transfer system, whereas other alkalinity-tolerant rhizobacteria have the ability to produce indole acetic acid and ACC (Msimbira and Smith, 2020).

Nutrient deficiency

Under nutrient stress, plants interact with rhizosphere microbes and release phytochemicals that aid in nutrient solubilization (Jones and Darrah, 1994). Phenolics are important root-exuded phytochemicals that induce solubilization and release iron (Fe), phosphorus (P), and other nutrients, thereby helping plants improve nutrient absorption (Amb and Ahluwalia, 2016). Changes in the abundance and diversity of specific microbial taxa, with subsequent alterations in microbial functional traits, are observed under nutrient-deficient conditions (Zhang et al., 2022). These changes can have profound effects on rice health and nutrient acquisition capability. Microbes can enhance the nutrient absorption of rice; for example, *Sphingomonas* spp., isolated from rice seeds and roots, facilitate nitrogen (N) fixation (Xie and Yokota, 2006; Videira et al., 2009). Ectomycorrhizal fungi release phytohormones that promote plant root development (Vayssières et al., 2015), and blue-green algae (Cyanobacteria) improve soil fertility via N fixation and alkaline soil reclamation (Dhar et al., 2007; Sahu et al., 2012).

Heavy metals

Heavy metal contamination affects the composition and diversity of the rice phytobiome. Studies have revealed that heavy metals can alter the structure of microbial communities associated with the roots, rhizosphere, and phyllosphere of rice. For instance, cadmium (Cd) pollution significantly decreased microbial diversity in the rhizosphere of rice plants (Hou et al., 2018). Microbes with high pollutant tolerance or degradation capabilities are likely to be recruited to the rhizosphere by the rice plant, thereby mitigating the detrimental effects of pollutants on microbial communities and plant growth (Li et al., 2022b). Certain bacterial and fungal species possess mechanisms to mitigate heavy metal toxicity via metal sequestration, enzymatic detoxification, and phytohormone modulation. The rhizosphere bacteria *Pantoea* sp. were found to reduce arsenic (As) uptake in rice (Elumalai et al., 2017), and bacteria from the genus *Sphingomonas* are capable of As redox transformation and detoxification in the rice ecosystem (Sultana et al., 2023). Cyanobacteria act as a biosorbent, reducing Cd accumulation and alleviating Cd toxicity (Kuang et al., 2016; Li et al., 2022a). Several bacterial taxa (e.g., *Bradyrhizobium*, *Bryobacter*, *Candidatus Solibacter*, *Geobacter*, *Gemmatimonas*, *Halingium*, *Sphingomonas*) showed a strong correlation with As and antimony contaminant fractions, indicating their potential for metabolizing these elements.

MANIPULATING THE RICE PHYTOBIOME FOR RICE IMPROVEMENT

Phytobiome manipulation constitutes a holistic approach that involves scrutinizing all components of the phytobiome as a mega dataset and deliberately applying various interventions to augment the benefits of the intricate interactions within the phytobiome ecosystem. Phytobiome manipulation aims to enhance nutrient uptake, bolster disease resistance, and optimize overall crop productivity through systematic and strategic measures. This approach extends to developing innovative management patterns designed to strengthen the adaptability of the phytobiome in anticipation of possible climate-change scenarios. By comprehensively examining the phytobiome mega dataset, we can reveal novel target genes and apply cutting-edge gene-editing techniques for crop improvement. Advanced molecular methods, such as metagenomics, meta-transcriptomics, meta-proteomics, and metabolomics, can facilitate the comprehensive profiling of microbial communities, aid in identifying novel microorganisms with desirable functional properties, and deploy tailored microbial formulations to optimize rice-microbe interactions. This approach entails systematic isolation, characterization, and evaluation of microbial strains, considering their physiological, genetic, and ecological attributes. Identifying novel microorganisms with potential benefits to rice improvement could expand the repertoire of microbial consortia applicable for biofertilization and biocontrol.

Manipulating the rice phytobiome for crop improvement and resilience under climate change could be accomplished by engineering the rice plant to enhance the capture of beneficial effects of the macro-/microbiome populations, including the improved release of hormones and signaling pathways that promote rice growth, development, and stress response. Alternatively, the positive effects of macro-/microbiome populations could be augmented by manipulating their populations and releasing beneficial organisms and the substances/hormones they produce into the rice phytobiome. Some notable examples are discussed below.

Altering rice stress-signaling pathways and interactions

The primary consideration in manipulating the rice phytobiome for rice fitness and climate-change resilience is modulating stress responses to maximize resistance while minimizing costs due to pleiotropic effects. Altering rice stress-signaling pathways could be one such method. Genes upregulated in each step of the key hormone pathways for growth and stress response are being identified. They could be modified by genetic engineering or gene editing to improve rice fitness and enhance stress tolerance. Several studies have demonstrated that the overexpression of key stress-related genes conferred improved rice tolerance to various abiotic stresses: *OsMYB6* and *OsDhn1* for both salt and drought stress (Kumar et al., 2014; Tang et al., 2019), *OsiSAP1* for water-deficit stress (Dansana et al., 2014), *OsTPP1* for salt and cold stress (Ge et al., 2008), and *OsPIN2* for better root growth and formation under nutrient (P) deficiency (Sun et al., 2019), among others. Genetic alterations can also be designed to improve interactions within the phytobiome, such as promoting symbiosis with beneficial microbes and enhancing the selection of rhizobial partners by host plants (Jain et al., 2023).

Exploiting the phyllosphere microbiome

The phyllosphere microbiome contributes to the cycling of nutrients, degradation and sequestration of pesticides and air pollution residues, and improvement of plant growth and health (Bashir et al., 2022); thus, it can enhance phytobiomes (Bashir et al., 2022). Beneficial endophytes are thought to be a novel source of biocontrol and biofertilizers for increasing crop yields (Mano and Morisaki, 2008; Gottel et al., 2011; Bertani et al., 2016; Sengupta et al., 2017). Rice-associated bacterial endophytes such as *Azoarcus* sp. and *Azospirillum* sp. stimulate plant growth and can be applied as endophyte inoculants to benefit rice productivity (Egener et al., 1999; Isawa et al., 2010; Yasuda

et al., 2022). Rice endophytic *Enterobacter* species also improve plant growth by enriching N and P supplies (Hardoim et al., 2013). *Methylobacterium* species that thrive on plant surfaces and use methanol released by plants via pectin demethylation encourage better seed ripening and promote seedling germination, growth of lateral roots, and general plant growth through the production of ACC deaminase, indole acetic acid, cytokinin, and siderophores (Madhaiyan et al., 2007; Chinnadurai et al., 2009; Senthilkumar et al., 2009; Tani et al., 2015). Indole acetic acid generated by *Bacillus*, *Pantoea*, *Stenotrophomonas*, *Achromobacter*, and *Exiguobacterium* bacteria encourages rice growth *in vitro*. Rice phyllosphere microbes such as fungi representing *Pestalotia*, *Alternaria*, and *Trichoderma* species (Naeimi et al., 2010) and bacteria such as *Bacillus pumilus* and *Erwinia* can also be used as biocontrol agents because of their antagonistic action against rice pathogens such as *Rhizoctonia solani*, *Xanthomonas oryzae*, and *Magnaporthe oryzae* (De Costa et al., 2006; Ilsan et al., 2015; Krishanti et al., 2015; Thapa et al., 2018). In addition, leaf actinomycetes species from *Lentzea*, *Streptomyces*, *Gordonia*, and *Saccharothrix* display antifungal activity against rice blast fungus (Harsonowati et al., 2017).

Rhizosphere engineering

Rhizosphere engineering, which involves targeted interventions to enhance plant–microbe interactions in the rhizosphere and thus improve nutrient uptake, disease resistance, and overall crop productivity, offers an excellent approach for manipulating the rice phytobiome. Inoculation with rhizosphere microbes, including PGPR and mycorrhizal fungi, has been shown to improve rice growth and health (Bao et al., 2022). PGPR strains such as *Azospirillum*, *Bacillus*, and *Pseudomonas* promote rice growth, enhance nutrient availability, and induce systemic resistance against pathogens (Isawa et al., 2010; Sivasakthi et al., 2014). A rice rhizosphere bacterium, *Pantoea* sp., encouraged healthy rice growth and improved the oxidizing potential of the rhizosphere (Lakshmanan et al., 2016). Similarly, mycorrhizal fungi, such as *Glomus* and *Rhizophagus*, improve rice root architecture and growth, nutrient uptake, and tolerance of abiotic stresses (Gutjahr et al., 2009).

Enhancing the production of root exudates and allelochemicals

Root exudates play a crucial role in shaping the composition and activity of the rice rhizosphere microbiome. Various approaches have been explored to manipulate root exudation profiles to favor the proliferation of beneficial microbes. For instance, overexpression of genes involved in the biosynthesis of specific root exudate compounds, such as phenolic acids and flavonoids, has been shown to attract beneficial rhizobacteria and suppress pathogens in rice (Wang et al., 2019; Du et al., 2021). Allelochemicals can be used as herbicides and growth stimulants, and crop genomes can also be modified for enhanced allelochemical production (Einhellig, 1995). For instance, a gene (*OsPAL2-1*) that regulates rice allelopathy by controlling the synthesis of phenolic acid allelochemicals has been identified (Li et al., 2020). This compound promotes the population of *Myxococcus xanthus* and produces another allelochemical, quercetin, that inhibits the germination and growth of a target weed. However, although promising, these approaches must address research gaps in understanding rice allelopathy, such as identifying allelopathic cultivars; discovering and isolating the allelopathic compounds; understanding the mechanism of action of each allelochemical; and evaluating activity in the soil, environmental interactions, and chemical communications between the rice plant and microorganisms that influence rice. Moreover, ecological risks, such as safety to humans and the ecosystem, crop productivity, and cost-benefit ratio, should be considered before genetically modifying crops to have enhanced allelopathic traits (Amb and Ahluwalia, 2016). The allelochemical target sites are crucial to breeding, as they determine which chemical to enhance in order to achieve the desired traits.

Conventional manipulation

Conventional manipulation of the rice phytobiome involves cultural and soil management practices such as organic matter amendments, cover cropping, and alternate wetting and drying, which can significantly influence the rice rhizosphere microbiome and plant fitness. Incorporating organic matter amendments, such as compost and animal or green manure, improves soil fertility, increases microbial biomass, and enhances nutrient availability in the rice rhizosphere (Das et al., 2017; Wang et al., 2017a; Cui et al., 2018). Integrating cover crops, such as legumes and grasses, improves soil structure, increases microbial biodiversity, and enhances nutrient cycling in rice systems (Kim et al., 2013). Furthermore, optimizing water management with alternate wetting and drying influences the composition of the rhizosphere microbial community and the nutrient dynamics of rice (Watanabe et al., 2021).

Manipulating one or more components of the rice phytobiome will create a domino effect that could positively or negatively affect the whole system, including multi-kingdom composition, interactions, signaling, and processes. A systems-level understanding of the rice phytobiome is necessary to determine whether other community members can co-opt, modify, or eliminate signals. Thus, a system-wide approach should be considered in manipulating the rice phytobiome to harness all possible beneficial effects of each component with minimal disruption to the whole system. To realize this goal, the next challenge will be to decode and re-program the information for rice improvement using advanced and sophisticated tools.

NOVEL APPROACHES FOR RE-PROGRAMMING THE RICE PHYTOBIOME

The rice phytobiome is a multilayer system

Our understanding of biological systems has shifted in the last 20 years. The idea that organisms are discrete entities with boundaries is obsolete, and in its place is the idea of the holobiont, which describes individual phenotypes as the result of interactions between the host and related microbial species (Simon et al., 2019). The biological processes of the host and the function of microbiomes are equally important in this circumstance. The term microbiome was recently broadened to encompass all microbial populations inhabiting a particular environment, their characteristics, and their interactions by a team of multidisciplinary specialists in the area (Whipps et al., 1988; Berg et al., 2020). Plants are naturally included in the interpretation of biological communities as holobionts

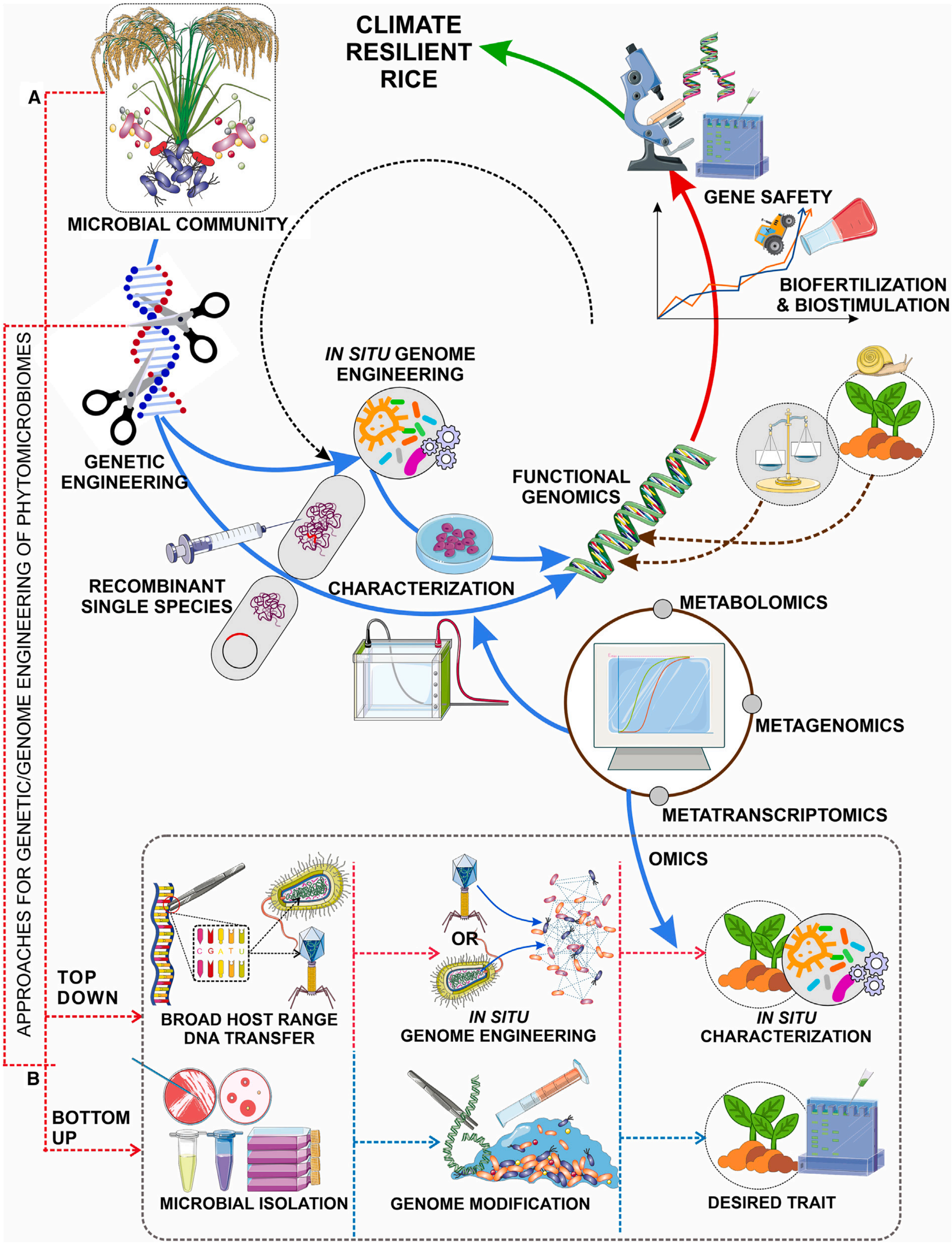

Figure 3. Schematic of synthetic biology–enabled microbiome engineering steps to achieve climate-resilient rice and their approaches.

(A) Numerous microorganisms with a variety of functions are linked to plants. Certain microorganisms (first group) can benefit their host plants by PGP; the second group can strongly colonize them. It is crucial to gather both groups at the first stage. The first group supplies PGP genes and pathways,

(legend continued on next page)

(Vandenkoornhuyse et al., 2015). The collective knowledge across all plant microbial domains has defined their critical role: distinct communities are linked to enhance plant growth via nutrient fixation, defense against biotic and abiotic stressors, modifications in secondary metabolite composition, and clearly defined growth stages (Berg et al., 2016). Studies of plant microbiomes have recently used highly reduced communities called SynCom to dissect their effects on plants (Lebeis et al., 2015), and these have been proposed for field use to promote plant growth in agricultural contexts (De Souza et al., 2020). Plants dynamically and spatially regulate microbiota composition in response to environmental stresses, leading to the "cry for help" hypothesis supported by the discovery of plant genetic regulators that integrate stress signaling and microbiota shaping. Insights into the key regulators and pathways of stress-alleviating microbiota assembly could aid in designing crops that dynamically recruit beneficial microbiota under stress conditions (Wang and Song, 2022).

In the context of plant pathogenesis, emerging evidence highlights the role of the pathobiome, the disease-contributing component within the plant microbiome. In this paradigm, disease onset and progression involve complex pathobiome-coordinated interactions at multiple scales and are not simply dominated by a single pathogen. Although an understanding of pathobiome-influenced plant pathogenesis is lacking, in-depth information on the composition and molecular mechanisms of the pathobiome offers a promising solution to improving disease-prevention strategies (Lv et al., 2023).

Plant microbiome engineering in the phytobiome ecosystem

Communities of microbes associated with plants, known as plant microbiomes (a large part of the phytobiome), show robust potential to offer affordable and long-lasting solutions to the agricultural challenges caused by climate change. Microorganisms have access to a variety of ecological niches within plants (Compant et al., 2019; Levy et al., 2018), and PGP traits are also provided by microorganisms (Shelake et al., 2019). Numerous approaches are available for researchers to further understand the relationships between plant microbiomes through meta-omic investigations and computational tools (Mitter et al., 2016). Numerous PGP microorganisms have been identified and isolated, and several are recognized as valuable biostimulants, biocontrol agents, and fertilizers. Nonetheless, there has not been much success using PGP microorganisms in domains intended for commercial use (Jäderlund et al., 2008; Bloch et al., 2020) because plant microbiomes are highly complex, diverse, and dynamic systems. Such tactics are probably going to be used only in certain contexts. Microbiome engineering based on synthetic biology is becoming more widely acknowledged as a means of providing host plants with PGP benefits to overcome these constraints (Mueller and Sachs, 2015). One such method enables the selection of microorganisms in a lab setting on the basis of their capacity to colonize plants, particularly regarding how effectively they can provide PGP benefits. Researchers can introduce these bacteria to certain plant species and sites (e.g., roots, leaves) at distinct growth and developmental phases under various environmental conditions. Furthermore, the designed microbiomes can consolidate many PGP features. We next examine current developments in synthetic biology and strain engineering to make microbiome engineering more suitable for rice climate-resilience applications and suggest approaches to limit environmental implications, as shown in Figure 3A.

Plant microbiome engineering techniques

Plant microbiomes can be developed in a top-down or bottom-up approach, as shown in Figure 3B. The bottom-up method separates ambient microbiomes from microbes linked to specific plant species, strains, or organs (Rodrigues et al., 2018; Toju et al., 2018). These essential microorganisms are reassembled as consortia after being genetically engineered to exhibit desired features (Vorholt et al., 2017). The modified strains are then inoculated into plants, allowing them to recolonize their hosts successfully. Horizontal gene transfer is used in the top-down method to impart desirable characteristics to various hosts *in situ*. Using mobile genetic elements (MGEs) is one top-down approach that enables a comprehensive study of PGP features. MGEs transfer and integrate foreign genes into a randomly selected subpopulation of microbiomes. Creating bacteriophage (phage) systems to design or eradicate certain species within populations, allowing their functions to be investigated, is another top-down strategy.

Synthetic biology of the plant microbiome to counter future challenges

PGP microbes have an extremely high potential to become game-changing actors in sustainable agriculture in the face of climate-change challenges. Still, success has been variable, likely because of varying environmental conditions, poor microbial colonization, and limited persistence in the phytobiome (Ke et al., 2021). These restrictions could be addressed by genome/genetic engineering of resilient root colonizers or by colonizing vast subpopulations of plant microbiomes. Non-model microbes are engineered even at the *in situ* level in subpopulations through emerging novel progress in synthetic biology. The following action evaluates the perseverance of genetically modified microorganisms (GMMs), the efficacy of engineered PGP advantages, and their association with plants in uncontrolled conditions before commercial adoption for rice productivity and disease control. Specifically, the environmental effects of field treatments that use GMMs must be assessed over an extended period of time.

together with switches and sensors to regulate gene expression. To impart designed PGP features to host plants, the second group could offer the best framework.

(B) Methods for phytobiome genetic/genome engineering. Two methods exist for genetic engineering or genome engineering of the phytobiome. Using a bottom-up methodology, plant-associated microorganisms are isolated, individual strains are manipulated to confer desired features, and plants are injected with the modified strains. The top-down method introduces features into various hosts *in situ* by horizontal gene transfer. Omics technologies and accompanying equipment are then used to identify the host phenotypes.

CHARACTERIZATION OF MICROBIOME DATA THROUGH ML AND DL APPROACHES

A large subset of artificial intelligence (AI) techniques called ML and DL uses massive datasets to perform pattern prediction, classification, and recognition (Tarca et al., 2007). ML has been used in microbiome research to tackle problems such as the classification of microbial features (determination of diversity, distribution, and abundance), phenotyping (prediction of host phenotype or an environment), tracking of any possible modifications to the composition of a target microbiome, and examination of the intricate chemical and physical relationships among the constituents of the microbiome (Gupta and Gupta, 2021; Marcos-Zambrano et al., 2021), as shown in Supplemental Table 2, which includes a few instances of each of these tasks.

Types of microbiome data

Developments in HTS and omics have made it possible to thoroughly describe the microbiome and create large-scale microbiome datasets (Jiang et al., 2019; Liu et al., 2021), although only a small portion of microbial species can be fully characterized using conventional isolation and cultivation methods (Lewis et al., 2021). Metagenome sequencing and amplicon technology are the most widely used techniques for microbiome analysis. Reads from commonly used taxonomic marker genes such as the ITS region (Schoch et al., 2012) or the evolutionarily conserved 16S rRNA gene (Weisburg et al., 1991) are used to characterize samples through the amplicon approach. Operational taxonomic units (OTUs) are clusters formed by roughly demarcating bacterial taxa on the basis of a predetermined identity threshold, typically 97% similarity (Schloss and Handelsman, 2005). With improvements in sequencing accuracy, OTUs have been replaced by ASVs (amplicon sequence variants), which require 100% identity. Because ASVs lack an arbitrary dissimilarity criterion and are produced using a de-noising technique, they enable the resolution of even uncommon (rare) community members (Callahan et al., 2017). However, shotgun metagenomics uses nonspecific sequencing to thoroughly catalog every genome in a sample (Gilbert and Dupont, 2011). Shotgun metagenomic readings can be curated for taxonomic or functional annotation by aligning them with databases using various algorithms (Liu et al., 2021). Recent developments even allow for the identification of the virome and open the door to more comprehensively characterize and reveal the microbiome through a shotgun database (Johansen et al., 2022). These methods result in feature tables in which each cell indicates the abundance or existence of a certain function or taxon for each sample. Disagreement exists about which kind of profile (functional or taxonomic) offers a greater capacity for discrimination in downstream analysis (Langille et al., 2013; Xu et al., 2014; Ning and Beiko, 2015). Either way, it is necessary to recognize the peculiarities and difficulties associated with these kinds of data. A feature table is compositional in the first place. Component relations are described using compositional data. In compositional data, they are totally arbitrary and each part is dependent (Aitchison, 1982; Quinn et al., 2018). Feature tables are also typically high dimensional (with more features per sample) and sparse (with an excessive number of zero counts). This feature exposure makes downstream analysis susceptible to the dimensionality curse. The dimensionality curse has two aspects: generalization to different datasets is weakened by a comparatively small number of samples, and an extreme number of features drives up computational expenses (Liu and Bellet, 2019). Various approaches are used to handle microbiome data. Statistical techniques such as computing component ratios (Greenacre, 2017), the staying-in-the-simplex approach (Mateu-Figueras et al., 2011), and log-ratio transformations (Aitchison, 1982) have been devised because typical distance and association measures are not suitable for compositional data. Because sparsity cannot be managed by traditional log-ratio transformation techniques, the data are frequently imputed, with pseudo-counts typically used in place of zeros (Costea et al., 2014). However, feature extraction and selection methods can assist in overcoming the drawbacks of dimensionality. The process of feature selection involves identifying non-redundant features and the best subspace of pertinent features (Peng et al., 2005; Ditzler et al., 2015). However, feature extraction builds a compressed version of the input features in an effort to decrease the dimensionality of the dataset. Pre-processing procedures are necessary because of the unique characteristics of microbiome data, and these procedures have a significant effect on differential feature analysis, which undoubtedly has an impact on the performance of ML techniques (Weiss et al., 2017; Nearing et al., 2022).

Traditional ML methods

For microbiome data, support vector machines, random forests (RFs), and linear regression models perform well among the standard ML techniques (Statnikov et al., 2013; Pasolli et al., 2016). In more recent research, however, the latter (Hernández Medina et al., 2022) has been reduced to benchmarking and has become obsolete. The interpretation of linear regression techniques, such as elastic nets and lasso, is simple because they represent an output (e.g., a phenotype) as a linear combination of inputs. Decision trees, which resemble flowcharts and are created by selecting which groups in a dataset to divide it into, are aggregated using RFs (Hernández Medina et al., 2022). An RF that outperforms a single tree can be created by generating many trees using randomly chosen feature subsets (Ho, 1995). Forecasting maize production (Chang et al., 2017) and resolving the symbiont density of sponges (Moitinho-Silva et al., 2017) have been accomplished using RFs with microbiome census data.

Dimensionality reduction in microbiome data

Unsupervised ordination techniques minimize dimensionality to simplify data for human comprehension. These methods are suitable for generating projections or visualizations; they provide a compressed representation of the input data by calculating a non-linear or linear combination of the current characteristics. Popular techniques for contrasting and visualizing microbial communities include linear approaches such as principal-coordinate analysis and principal-component analysis, which have been used to determine the geographic origin or habitat of microbial samples (Costello et al., 2009; Porras et al., 2021). Techniques such as uniform manifold approximation and projection and t-distributed stochastic neighbor embedding accurately detect and highlight non-linear and local connections

in sophisticated datasets of a target microbiome; nonetheless, regulating them is a difficult task (Xu et al., 2014, 2020; Kostic et al., 2015; Armstrong et al., 2021).

DL approaches

A family of ML methods called DL uses several artificial neural network designs. To provide precise insights and forecasts, DL models can identify intricate patterns such as images, text, and other types of data. Nodes (also referred to as units or neurons) are essential to DL models and are operations that modify inputs and send outputs to the next (other) nodes. The network that is created by the connections among nodes is made up of several layers that can be linked together and arranged in various architectures or layouts. The fully connected neural network (FCNN) is the most basic neural network design, in which all nodes from a given layer are entirely linked to all nodes from the layer below. Using raw metagenomics count data, researchers have used this architecture to predict the host phenotype, which can result in higher classification accuracy across many datasets compared with conventional approaches (Lo and Marculescu, 2018). Although the FCNN works well on its own, it is typically the fundamental component of more intricate architectures.

CONSIDERATIONS FOR WORKFLOW

Input from microbial omics

From collecting samples to the bioinformatics pipeline, microbial omics errors may limit or detour the information obtained (Kaster and Sobol, 2020). In most cases, there should be a trade-off in the experimental design, for which recommendations have been provided (Franzosa et al., 2015). Resolution in metataxonomics is often restricted to the genus level. Even so, the omics input for supervised ML (SML) is the most frequently employed, and the feature set is composed of relative OTU abundances (Janßen et al., 2021; Kim and Oh, 2021). Nevertheless, the use of OTUs might overlook significant taxonomic groupings and is intrinsically restrictive in terms of maintaining community information. ASVs (Callahan et al., 2017) that have more biological significance have been developed, but metataxonomic studies notably lack ASVs. Given that ASVs provide a more precise foundation for taxonomy, it will be intriguing to observe how their use affects ML outcomes in the future. Although metagenomics is extremely sensitive for low-abundance taxa, it is rarely used for SML and comes with extra expenditures that could restrict ML options and samples (Callahan et al., 2017). Metagenomic methods do not consistently outperform the more economical metataxonomic method (Xu et al., 2014).

Selection of the ML model

An extensive set of SML tools is available, and each has unique benefits and drawbacks (Goodswen et al., 2021). Users must make trade-offs between interpretability, learning performance, computational costs, data needs, and simplicity of implementation because no architecture works well in all environmental application scenarios (Ghannam and Techtmann, 2021). Choosing a collection of architectures at the outset can help to guarantee the accomplishment of research objectives. RF is a popular option for microbial omics-driven SML because of its high interpretability, ease of implementation, and learning capability (Ghannam and Techtmann, 2021). DL techniques (multi-layered architectures) perform well for super-complicated tasks or situations in which information is scarce because they can self-learn the feature set (Christin et al., 2019). However, DL demands massive data and thousands of samples. It is associated with high processing costs and limited interpretability of the underlying model—the "black box" effect. As a result, although up and coming, DL techniques for environmental omics are still limited (Figure 4).

Feature engineering

Selection and engineering of features are pivotal steps for producing SML-based ecological models of significance. Limiting overfitting, cutting down on computational expenses, enhancing cross-study comparability, and improving generalized prediction accuracy across datasets are possible with reduced feature space (Ghannam and Techtmann, 2021). When reducing features for training, caution must be used because it is possible to overlook physiologically significant traits if abundance is the basis for feature selection. Optimizing feature selection in metataxonomic-based ML applications can be achieved using biologically driven feature-selection techniques (Oudah and Henschel, 2018) or embedded methodologies (Wang et al., 2017b). There is now an apparent deficiency in feature-selection techniques for functional feature sets. Given the compositional character of microbial omics datasets, caution is required when using conventional statistics, which might make assumptions about the underlying data (Gloor et al., 2017). Remembering that SML aims to enhance rather than replace traditional statistical modeling is crucial. Combining these two methods offers robust opportunities to use their benefits for environmental and phytobiome microbiology and monitoring predictions. There is still more work to be done in feature selection and engineering for multi-omics investigations as the systems levels increase in complexity.

Assessing data leakages

Data leakage, which refers to the unintentional use or impact of data during the training process, is a subtle but significant feature of ML. This frequently happens when the training characteristics conceal the outcome of the prediction from themselves, leading to an overestimation of the validation performance of the model (Chiavegatto Filho et al., 2021). Because of how nuanced this might be, preventing data leaks is complex; it needs to be assessed case by case (Wirbel et al., 2021) and to include (1) target label-influenced data filtering and (2) the division of dependent data between validation and training sets. Using an outside-produced test dataset might be beneficial for further validation tests. However, data leaking is rarely addressed in microbial omics articles that use SML (Wirbel et al., 2021).

ADDRESSING CLIMATE CHANGE WITH SML OF PHYTOBIOME-RELATED MICROBIAL OMICS DATA

Understanding the interactions and activities among microbes, phytobiomes, and ecosystems is crucial for their incorporation into ecological models and biotechnologies to mitigate climate change. The production of high-resolution spatiotemporal dynamics data and the integration of several omics datasets could

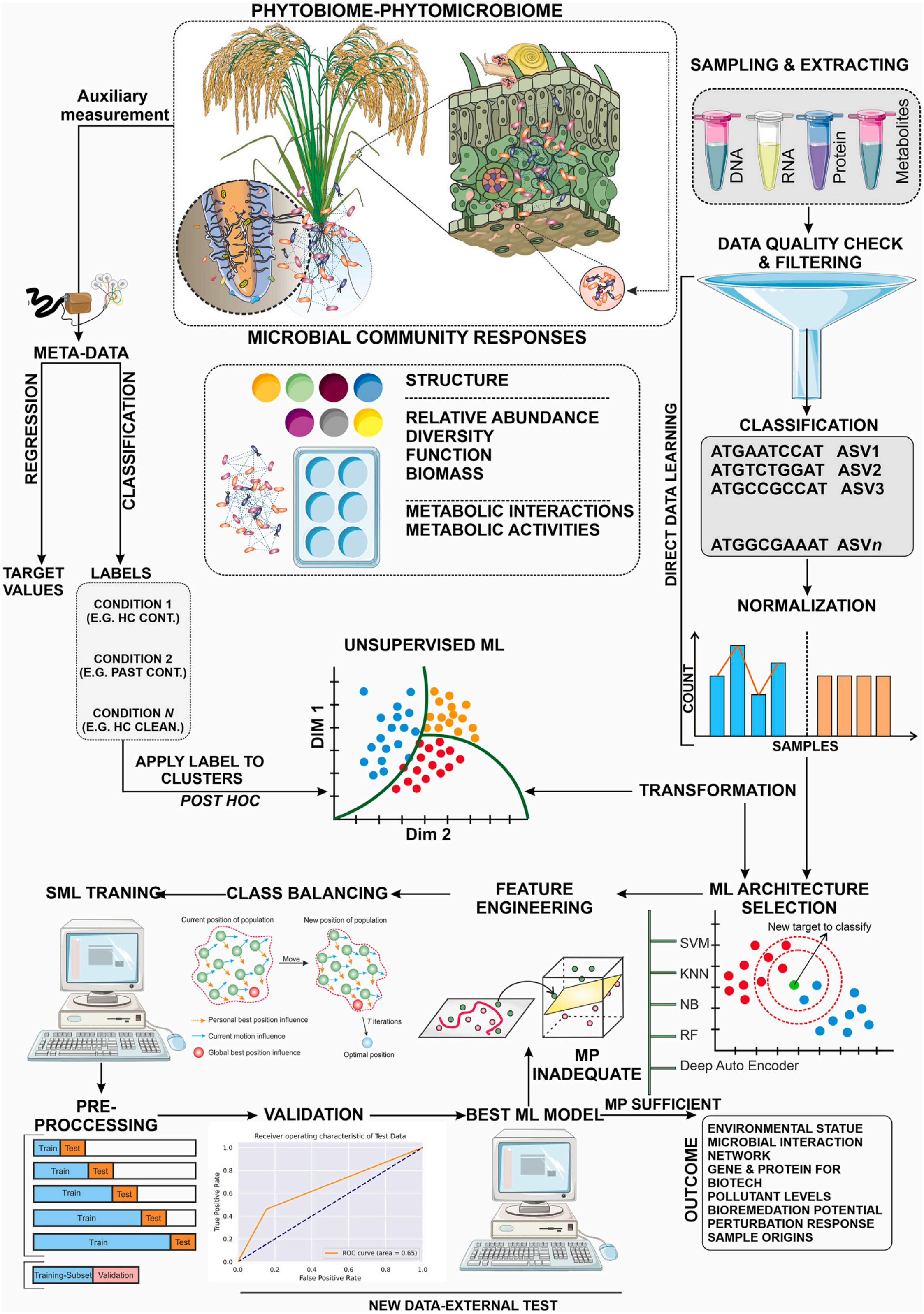

Figure 4. The interaction and interface in a general workflow to use ML approaches in encounters with rice phytobiome microbiome omics datasets.

enhance the precision of forecasting models and offer significant insights into the underlying reactions of molecular processes to climate change (Herold et al., 2020; Layton and Bradbury, 2022). In conjunction with their ubiquitous nature, the essential functions of phytobiome microbial communities provide us with a comprehensive framework for prospective microbiological instruments that could be used to understand, monitor, predict, and perhaps lessen the fundamental repercussions of global climate change.

CONCLUDING REMARKS AND FUTURE PERSPECTIVES

The rice phytobiome network, a complex assemblage of micro- and macroorganisms that interact with rice and its environment, is crucial for adapting to the challenges of climate change. The diverse organisms within the rice phytobiome have direct and indirect influences on rice plant health and nutrient acquisition, as well as overall rice ecosystem functioning; thus, it is imperative to understand the complex interactions within the rice phytobiome to maximize its potential for climate change adaptation. Among the leading actors in the rice phytobiome are the microbiome communities. Here, we conceptualized the rice phytobiome as an informative and integrated system with deeply hidden information and the plant microbiomes as a gigantic dataset with esoteric sophisticated information for adaption of rice to future challenges. Using novel, data-driven, and systems-level approaches, the rice phytobiome can be accurately re-programmed to resist climate change threats by developing prescriptive and predictive analytics for next-generation precision rice agricultural systems. AI approaches can handle massive phytobiome data characterized by a mixture of several species, high dimensionality, and sparsity owing to a lack of comprehensive annotator expertise. ML techniques can be used to perform prediction tasks and statistical associations between phytobiome data and rice phenotypes. However, improved integration of multifaceted information on rice phytobiome data is required for DL techniques to forecast phytobiome attributes more accurately and thus improve rice for future climate-change challenges.

FUNDING

The author(s) declare that financial support was received for the authorship and/or publication of this article. This publication was funded by the International Rice Research Institute-Hybrid Rice Development Consortium and the AGGRi Alliance project ‘‘Accelerated Genetic Gains in Rice Alliance’’ by the Bill and Melinda Gates Foundation through grant no. OPP1194925- INV 008226.

ACKNOWLEDGMENTS

No conflict of interest declared.

AUTHOR CONTRIBUTIONS

S.M.H.K., N.G.D. and J.A., as the main colleagues, made the main contributions to design and manuscript writing. J.A., as executive of the project, was involved in project approval and manuscript writing. S.M.H.K., N.G.D., E.V., V.S., and J.A. cooperated in revising the manuscript. S.M.H.K., N.G.D., and J.A. cooperated in the final editing of the manuscript. All authors contributed to revising the manuscript. All authors read and approved the final manuscript.

SUPPLEMENTAL INFORMATION

Supplemental information is available at *Plant Communications Online*.

Received: April 27, 2024
Revised: August 7, 2024
Accepted: September 2, 2024
Published: September 3, 2024

REFERENCES

Abdul Rahman, N.S.N., Abdul Hamid, N.W., and Nadarajah, K. (2021). Effects of abiotic stress on soil microbiome. Int. J. Mol. Sci. **22**:9036. https://doi.org/10.3390/ijms22169036.

Aggarwal, R., Subramanyam, S., Zhao, C., Chen, M.-S., Harris, M.O., and Stuart, J.J. (2014). Avirulence effector discovery in a plant galling and plant parasitic arthropod, the Hessian fly (Mayetiola destructor). PLoS One **9**:e100958. https://doi.org/10.1371/journal.pone.0100958.

Ahuja, I., Kissen, R., and Bones, A.M. (2012). Phytoalexins in defense against pathogens. Trends Plant Sci. **17**:73–90. https://doi.org/10.1016/j.tplants.2011.11.002.

Aitchison, J. (1982). The statistical analysis of compositional data. J. Roy. Stat. Soc. B **44**:139–160. https://doi.org/10.1111/j.2517-6161.1982.tb01195.x.

Akatsuka, T., Kodama, O., Sekido, H., Kono, Y., and Takeuchi, S. (1985). Novel phytoalexins (oryzalexins A, B and C) isolated from rice blast leaves infected with Pyricularia oryzae. Part I: Isolation, characterization and biological activities of oryzalexins. Agricultural and biological chemistry **49**:1689–1694. https://doi.org/10.1080/00021369.1985.10866951.

Alborn, H.T., Hansen, T.V., Jones, T.H., Bennett, D.C., Tumlinson, J.H., Schmelz, E.A., and Teal, P.E.A. (2007). Disulfooxy fatty acids from the American bird grasshopper Schistocerca americana, elicitors of plant volatiles. Proc. Natl. Acad. Sci. USA **104**:12976–12981. https://doi.org/10.1073/pnas.0705947104.

Amb, M., and Ahluwalia, A. (2016). Allelopathy: potential role to achieve new milestones in rice cultivation. Rice Sci. **23**:165–183. https://doi.org/10.1016/j.rsci.2016.06.001.

Angel, R., Kammann, C., Claus, P., and Conrad, R. (2012). Effect of long-term free-air CO2 enrichment on the diversity and activity of soil methanogens in a periodically waterlogged grassland. Soil Biol. Biochem. **51**:96–103. https://doi.org/10.1016/j.soilbio.2012.04.010.

Armstrong, G., Martino, C., Rahman, G., Gonzalez, A., Vázquez-Baeza, Y., Mishne, G., and Knight, R. (2021). Uniform manifold approximation and projection (UMAP) reveals composite patterns and resolves visualization artifacts in microbiome data. mSystems **6**:e0069121, e00691-00621. https://doi.org/10.1128/msystems.00691-21.

Asaf, S., Numan, M., Khan, A.L., and Al-Harrasi, A. (2020). Sphingomonas: from diversity and genomics to functional role in environmental remediation and plant growth. Crit. Rev. Biotechnol. **40**:138–152. https://doi.org/10.1080/07388551.2019.1709793.

Avanci, N.C., Luche, D.D., Goldman, G.H., and Goldman, M.H.S. (2010). Jasmonates are phytohormones with multiple functions, including plant defense and reproduction. Genet. Mol. Res. **9**:484–505. https://doi.org/10.1093/jxb/erw495.

Badri, D.V., and Vivanco, J.M. (2009). Regulation and function of root exudates. Plant Cell Environ. **32**:666–681. https://doi.org/10.1111/j.1365-3040.2009.01926.x.

Bao, X., Zou, J., Zhang, B., Wu, L., Yang, T., and Huang, Q. (2022). Arbuscular mycorrhizal fungi and microbes interaction in rice mycorrhizosphere. Agronomy **12**:1277. https://doi.org/10.3390/agronomy12061277.

Barker, D.G., Chabaud, M., Russo, G., and Genre, A. (2017). Nuclear Ca2+ signalling in arbuscular mycorrhizal and actinorhizal endosymbioses: on the trail of novel underground signals. New Phytol. **214**:533–538. https://doi.org/10.1111/nph.14350.

Bashir, I., War, A.F., Rafiq, I., Reshi, Z.A., Rashid, I., and Shouche, Y.S. (2022). Phyllosphere microbiome: diversity and functions. Microbiol. Res. **254**:126888. https://doi.org/10.1016/j.micres.2021.126888.

Berendsen, R.L., Vismans, G., Yu, K., Song, Y., de Jonge, R., Burgman, W.P., Burmølle, M., Herschend, J., Bakker, P.A.H.M., and Pieterse, C.M.J. (2018). Disease-induced assemblage of a plant-beneficial bacterial consortium. The ISME journal **12**:1496–1507. https://doi.org/10.1038/s41396-018-0093-1.

Berens, M.L., Wolinska, K.W., Spaepen, S., Ziegler, J., Nobori, T., Nair, A., Krüler, V., Winkelmüller, T.M., Wang, Y., Mine, A., et al. (2019). Balancing trade-offs between biotic and abiotic stress responses through leaf age-dependent variation in stress hormone cross-talk. Proc. Natl. Acad. Sci. USA **116**:2364–2373. https://doi.org/10.1073/pnas.1817233116.

Berg, G., Rybakova, D., Grube, M., and Köberl, M. (2016). The plant microbiome explored: implications for experimental botany. J. Exp. Bot. **67**:995–1002. https://doi.org/10.1093/jxb/erv466.

Berg, G., Rybakova, D., Fischer, D., Cernava, T., Vergès, M.-C.C., Charles, T., Chen, X., Cocolin, L., Eversole, K., Corral, G.H., et al. (2020). Microbiome definition re-visited: old concepts and new challenges. Microbiome **8**:103–122. https://doi.org/10.1186/s40168-020-00875-0.

Bertani, I., Abbruscato, P., Piffanelli, P., Subramoni, S., and Venturi, V. (2016). Rice bacterial endophytes: isolation of a collection, identification of beneficial strains and microbiome analysis. Environ. Microbiol. Rep. **8**:388–398. https://doi.org/10.1111/1758-2229.12403.

Bloch, S.E., Clark, R., Gottlieb, S.S., Wood, L.K., Shah, N., Mak, S.-M., Lorigan, J.G., Johnson, J., Davis-Richardson, A.G., Williams, L., et al. (2020). Biological nitrogen fixation in maize: optimizing nitrogenase expression in a root-associated diazotroph. J. Exp. Bot. **71**:4591–4603. https://doi.org/10.1093/jxb/eraa176.

Boller, T., and Felix, G. (2009). A renaissance of elicitors: perception of microbe-associated molecular patterns and danger signals by pattern-recognition receptors. Annu. Rev. Plant Biol. **60**:379–406. https://doi.org/10.1146/annurev.arplant.57.032905.105346.

Callahan, B.J., McMurdie, P.J., and Holmes, S.P. (2017). Exact sequence variants should replace operational taxonomic units in marker-gene data analysis. The ISME journal **11**:2639–2643. https://doi.org/10.1038/ismej.2017.119.

Canarini, A., Kaiser, C., Merchant, A., Richter, A., and Wanek, W. (2019). Root exudation of primary metabolites: mechanisms and their roles in plant responses to environmental stimuli. Front. Plant Sci. **10**:157. https://doi.org/10.3389/fpls.2019.00157.

Canarini, A., Schmidt, H., Fuchslueger, L., Martin, V., Herbold, C.W., Zezula, D., Gündler, P., Hasibeder, R., Jecmenica, M., Bahn, M., et al. (2021). Ecological memory of recurrent drought modifies soil processes via changes in soil microbial community. Nat. Commun. **12**:5308. https://doi.org/10.1038/s41467-021-25675-4.

Cartwright, D.W., Langcake, P., Pryce, R.J., Leworthy, D.P., and Ride, J.P. (1981). Isolation and characterization of two phytoalexins from rice as momilactones A and B. Phytochemistry **20**:535–537. https://doi.org/10.1016/S0031-9422(00)84189-8.

Challinor, A.J., Watson, J., Lobell, D.B., Howden, S.M., Smith, D.R., and Chhetri, N. (2014). A meta-analysis of crop yield under climate change and adaptation. Nat. Clim. Chang. **4**:287–291. https://doi.org/10.1038/NCLIMATE2153.

Chang, H.-X., Haudenshield, J.S., Bowen, C.R., and Hartman, G.L. (2017). Metagenome-wide association study and machine learning prediction of bulk soil microbiome and crop productivity. Front. Microbiol. **8**:519. https://doi.org/10.3389/fmicb.2017.00519.

CHANGE, G. (2019). Scientists' warning to humanity: microorganisms and climate change. Nat. Rev. Microbiol. **17**:569–586. https://doi.org/10.1038/s41579-019-0222-5.

Chiavegatto Filho, A., Batista, A.F.D.M., and Dos Santos, H.G. (2021). Data leakage in health outcomes prediction with machine learning. Comment on "Prediction of incident hypertension within the next year: Prospective study using statewide electronic health records and machine learning". J. Med. Internet Res. **23**:e10969. https://doi.org/10.2196/10969.

Chinnadurai, C., Balachandar, D., and Sundaram, S.P. (2009). Characterization of 1-aminocyclopropane-1-carboxylate deaminase producing methylobacteria from phyllosphere of rice and their role in ethylene regulation. World J. Microbiol. Biotechnol. **25**:1403–1411. https://doi.org/10.1007/s00425-007-0532-0.

Chou, C.-H. (1990). The role of allelopathy in agroecosystems: studies from tropical Taiwan. In Agroecology: Researching the Ecological Basis for Sustainable Agriculture (Springer), pp. 104–121. https://doi.org/10.1300/J144v04n02_01.

Chouhan, G.K., Mukherjee, A., Gaurav, A.K., Jaiswal, D.K., and Verma, J.P. (2021a). Plant-specific microbiome for environmental stress management: issues and challenges. New and future developments in microbial biotechnology and bioengineering, 69–89. https://doi.org/10.1016/B978-0-444-64325-4.00008-0.

Chouhan, G.K., Verma, J.P., Jaiswal, D.K., Mukherjee, A., Singh, S., de Araujo Pereira, A.P., Liu, H., Abd Allah, E.F., and Singh, B.K. (2021b). Phytomicrobiome for promoting sustainable agriculture and food security: opportunities, challenges, and solutions. Microbiol. Res. **248**:126763. https://doi.org/10.1016/j.micres.2021.126763.

Christin, S., Hervet, É., and Lecomte, N. (2019). Applications for deep learning in ecology. Methods Ecol. Evol. **10**:1632–1644. https://doi.org/10.1111/2041-210X.13256.

Chung, J., Goo, E., Yu, S., Choi, O., Lee, J., Kim, J., Kim, H., Igarashi, J., Suga, H., Moon, J.S., et al. (2011). Small-molecule inhibitor binding to an N-acyl-homoserine lactone synthase. Proc. Natl. Acad. Sci. USA **108**:12089–12094. https://doi.org/10.1073/pnas.1103165108.

Compant, S., Samad, A., Faist, H., and Sessitsch, A. (2019). A review on the plant microbiome: Ecology, functions, and emerging trends in microbial application. J. Adv. Res. **19**:29–37. https://doi.org/10.1016/j.jare.2019.03.004.

Costea, P.I., Zeller, G., Sunagawa, S., and Bork, P. (2014). A fair comparison. Nat. Methods **11**:359. https://doi.org/10.1038/nmeth.2897.

Costello, E.K., Lauber, C.L., Hamady, M., Fierer, N., Gordon, J.I., and Knight, R. (2009). Bacterial community variation in human body habitats across space and time. science **326**:1694–1697. https://doi.org/10.1126/science.1177486.

Cui, X., Zhang, Y., Gao, J., Peng, F., and Gao, P. (2018). Long-term combined application of manure and chemical fertilizer sustained higher nutrient status and rhizospheric bacterial diversity in reddish paddy soil of Central South China. Sci. Rep. **8**:16554. https://doi.org/10.1038/s41598-018-34685-0.

Dansana, P.K., Kothari, K.S., Vij, S., and Tyagi, A.K. (2014). OsiSAP1 overexpression improves water-deficit stress tolerance in transgenic rice by affecting expression of endogenous stress-related genes. Plant Cell Rep. **33**:1425–1440. https://doi.org/10.1007/s00299-014-1626-3.

Das, S., and Adhya, T. (2012). Dynamics of methanogenesis and methanotrophy in tropical paddy soils as influenced by elevated CO2 and temperature interaction. Soil Biol. Biochem. **47**:36–45. https://doi.org/10.1016/j.soilbio.2011.11.020.

Das, S., Jeong, S.T., Das, S., and Kim, P.J. (2017). Composted cattle manure increases microbial activity and soil fertility more than

composted swine manure in a submerged rice paddy. Front. Microbiol. **8**:1702. https://doi.org/10.3389/fmicb.2017.01702.

Davis, T.S., Crippen, T.L., Hofstetter, R.W., and Tomberlin, J.K. (2013). Microbial volatile emissions as insect semiochemicals. J. Chem. Ecol. **39**:840–859. https://doi.org/10.1007/s10886-013-0306-z.

De Costa, D., Pinto, M., Geethanjalee, H., and Dissanayake, N. (2006). Suppression of rice pathogens by phyllosphere associated microflora of different rice varieties in Sri Lanka. Trop. Sci. **46**:97–104. https://doi.org/10.1002/ts.63.

De Souza, R.S.C., Armanhi, J.S.L., and Arruda, P. (2020). From microbiome to traits: designing synthetic microbial communities for improved crop resiliency. Front. Plant Sci. **11**:1179. https://doi.org/10.3389/fpls.2020.01179.

Desmedt, W., Kudjordjie, E.N., Chavan, S.N., Zhang, J., Li, R., Yang, B., Nicolaisen, M., Mori, M., Peters, R.J., Vanholme, B., et al. (2022). Rice diterpenoid phytoalexins are involved in defence against parasitic nematodes and shape rhizosphere nematode communities. New Phytol. **235**:1231–1245. https://doi.org/10.1111/nph.18152.

Dhar, D.W., Prasanna, R., and Singh, B.V. (2007). Comparative performance of three carrier based blue green algal biofertilizers for sustainable rice cultivation. J. Sustain. Agric. **30**:41–50. https://doi.org/10.1300/J064v30n02_06.

Ding, L.-J., An, X.-L., Li, S., Zhang, G.-L., and Zhu, Y.-G. (2014). Nitrogen loss through anaerobic ammonium oxidation coupled to iron reduction from paddy soils in a chronosequence. Environ. Sci. Technol. **48**:10641–10647. https://doi.org/10.1021/es503113s.

Ditzler, G., Morrison, J.C., Lan, Y., and Rosen, G.L. (2015). Fizzy: feature subset selection for metagenomics. BMC Bioinf. **16**:358. https://doi.org/10.1186/s12859-015-0793-8.

Dresselhaus, T., and Hückelhoven, R. (2018). Biotic and abiotic stress responses in crop plants. MDPI **8**:267. https://doi.org/10.3390/agronomy8110267.

Du, B., Chen, R., Guo, J., and He, G. (2020). Current understanding of the genomic, genetic, and molecular control of insect resistance in rice. Mol. Breeding **40**:24–25. https://doi.org/10.1007/s11032-020-1103-3.

Du, Y., Chen, X., Guo, Y., Zhang, X., Zhang, H., Li, F., Huang, G., Meng, Y., and Shan, W. (2021). Phytophthora infestans RXLR effector PITG20303 targets a potato MKK1 protein to suppress plant immunity. New Phytol. **229**:501–515. https://doi.org/10.1111/nph.16861.

Edwards, J., Johnson, C., Santos-Medellín, C., Lurie, E., Podishetty, N.K., Bhatnagar, S., Eisen, J.A., and Sundaresan, V. (2015). Structure, variation, and assembly of the root-associated microbiomes of rice. Proc. Natl. Acad. Sci. USA **112**:E911–E920. https://doi.org/10.1073/pnas.141459211.

Egener, T., Hurek, T., and Reinhold-Hurek, B. (1999). Endophytic expression of nif genes of Azoarcus sp. strain BH72 in rice roots. Mol. Plant Microbe Interact. **12**:813–819. https://doi.org/10.1094/MPMI.1999.12.9.813.

Einhellig, F.A. (1995). Allelopathy: Current Status and Future Goals (ACS Publications). https://doi.org/10.1021/bk-1995-0582.ch001.

Elumalai, V., Brindha, K., and Lakshmanan, E. (2017). Human exposure risk assessment due to heavy metals in groundwater by pollution index and multivariate statistical methods: a case study from South Africa. Water **9**:234. https://doi.org/10.3390/w9040234.

Erb, M., Meldau, S., and Howe, G.A. (2012). Role of phytohormones in insect-specific plant reactions. Trends Plant Sci. **17**:250–259. https://doi.org/10.1016/j.tplants.2012.01.003.

Fitzpatrick, C.R., Salas-González, I., Conway, J.M., Finkel, O.M., Gilbert, S., Russ, D., Teixeira, P.J.P.L., and Dangl, J.L. (2020). The plant microbiome: from ecology to reductionism and beyond. Annu. Rev. Microbiol. **74**:81–100. https://doi.org/10.1146/annurev-micro-022620-014327.

Franzosa, E.A., Hsu, T., Sirota-Madi, A., Shafquat, A., Abu-Ali, G., Morgan, X.C., and Huttenhower, C. (2015). Sequencing and beyond: integrating molecular'omics' for microbial community profiling. Nat. Rev. Microbiol. **13**:360–372. https://doi.org/10.1038/nrmicro3451.

Fu, S.-F., Wei, J.-Y., Chen, H.-W., Liu, Y.-Y., Lu, H.-Y., and Chou, J.-Y. (2015). Indole-3-acetic acid: A widespread physiological code in interactions of fungi with other organisms. Plant Signal. Behav. **10**:e1048052. https://doi.org/10.1080/15592324.2015.1048052.

Fujita, D., Kohli, A., and Horgan, F.G. (2013). Rice resistance to planthoppers and leafhoppers. Crit. Rev. Plant Sci. **32**:162–191. https://doi.org/10.1080/07352689.2012.735986.

Fukumoto, K., Alamgir, K., Yamashita, Y., Mori, I.C., Matsuura, H., and Galis, I. (2013). Response of Rice to Insect Elicitors and the Role of Os JAR 1 in Wound and Herbivory-I nduced JA-I le Accumulation. J. Integr. Plant Biol. **55**:775–784. https://doi.org/10.1111/jipb.12057.

Gao, X., and Kolomiets, M.V. (2009). Host-derived lipids and oxylipins are crucial signals in modulating mycotoxin production by fungi. Toxin Rev. **28**:79–88. https://doi.org/10.1080/15569540802420584.

Ge, L.-F., Chao, D.-Y., Shi, M., Zhu, M.-Z., Gao, J.-P., and Lin, H.-X. (2008). Overexpression of the trehalose-6-phosphate phosphatase gene OsTPP1 confers stress tolerance in rice and results in the activation of stress responsive genes. Planta **228**:191–201. https://doi.org/10.1007/s00425-008-0729-x.

Ghannam, R.B., and Techtmann, S.M. (2021). Machine learning applications in microbial ecology, human microbiome studies, and environmental monitoring. Comput. Struct. Biotechnol. J. **19**:1092–1107. https://doi.org/10.1016/j.csbj.2021.01.028.

Gilbert, J.A., and Dupont, C.L. (2011). Microbial metagenomics: beyond the genome. Ann. Rev. Mar. Sci **3**:347–371. https://doi.org/10.1146/annurev-marine-120709-142811.

Girard, M., Luis, P., Valiente Moro, C., and Minard, G. (2023). Crosstalk between the microbiota and insect postembryonic development. Trends Microbiol. **31**:181–196. https://doi.org/10.1016/j.tim.2022.08.013.

Glazebrook, J. (2005). Contrasting mechanisms of defense against biotrophic and necrotrophic pathogens. Annu. Rev. Phytopathol. **43**:205–227. https://doi.org/10.1146/annurev.phyto.43.040204.135923.

Gloor, G.B., Macklaim, J.M., Pawlowsky-Glahn, V., and Egozcue, J.J. (2017). Microbiome datasets are compositional: and this is not optional. Front. Microbiol. **8**:2224. https://doi.org/10.3389/fmicb.2017.02224.

Goodswen, S.J., Barratt, J.L.N., Kennedy, P.J., Kaufer, A., Calarco, L., and Ellis, J.T. (2021). Machine learning and applications in microbiology. FEMS Microbiol. Rev. **45**:fuab015. https://doi.org/10.1093/femsre/fuab015.

Gottel, N.R., Castro, H.F., Kerley, M., Yang, Z., Pelletier, D.A., Podar, M., Karpinets, T., Uberbacher, E., Tuskan, G.A., Vilgalys, R., et al. (2011). Distinct microbial communities within the endosphere and rhizosphere of Populus deltoides roots across contrasting soil types. Appl. Environ. Microbiol. **77**:5934–5944. https://doi.org/10.1128/AEM.05255-11.

Gough, C., and Cullimore, J. (2011). Lipo-chitooligosaccharide signaling in endosymbiotic plant-microbe interactions. Mol. Plant Microbe Interact. **24**:867–878. https://doi.org/10.1094/MPMI-01-11-0019.

Greenacre, M. (2017). Towards a pragmatic approach to compositional data analysis.

Großkinsky, D.K., van der Graaff, E., and Roitsch, T. (2012). Phytoalexin transgenics in crop protection—Fairy tale with a happy

end? Plant Sci. **195**:54–70. https://doi.org/10.1016/j.plantsci.2012.06.008.

Gupta, M.M., and Gupta, A. (2021). Survey of artificial intelligence approaches in the study of anthropogenic impacts on symbiotic organisms–a holistic view. Symbiosis **84**:271–283. https://doi.org/10.1016/j.neucom.2023.126895.

Gutjahr, C., Casieri, L., and Paszkowski, U. (2009). Glomus intraradices induces changes in root system architecture of rice independently of common symbiosis signaling. New Phytol. **182**:829–837. https://doi.org/10.1111/j.1469-8137.2009.02839.x.

Hamonts, K., Trivedi, P., Garg, A., Janitz, C., Grinyer, J., Holford, P., Botha, F.C., Anderson, I.C., and Singh, B.K. (2018). Field study reveals core plant microbiota and relative importance of their drivers. Environ. Microbiol. **20**:124–140. https://doi.org/10.1111/1462-2920.14031.

Hardoim, P.R., Nazir, R., Sessitsch, A., Elhottová, D., Korenblum, E., van Overbeek, L.S., and van Elsas, J.D. (2013). The new species Enterobacter oryziphilus sp. nov. and Enterobacter oryzendophyticus sp. nov. are key inhabitants of the endosphere of rice. BMC Microbiol. **13**:164–213. https://doi.org/10.1186/1471-2180-13-164.

Harsonowati, W., Astuti, R.I., and Wahyudi, A.T. (2017). Leaf blast disease reduction by rice-phyllosphere actinomycetes producing bioactive compounds. J. Gen. Plant Pathol. **83**:98–108. https://doi.org/10.1007/s10327-017-0700-4.

Hartmann, A., Rothballer, M., Hense, B.A., and Schröder, P. (2014). Bacterial Quorum Sensing Compounds Are Important Modulators of Microbe-Plant Interactions (Frontiers Media SA). https://doi.org/10.3389/fpls.2014.00131.

Hatano, E., Saveer, A.M., Borrero-Echeverry, F., Strauch, M., Zakir, A., Bengtsson, M., Ignell, R., Anderson, P., Becher, P.G., Witzgall, P., et al. (2015). A herbivore-induced plant volatile interferes with host plant and mate location in moths through suppression of olfactory signalling pathways. BMC Biol. **13**:75. https://doi.org/10.1186/s12915-015-0188-3.

Haugwitz, M.S., Bergmark, L., Priemé, A., Christensen, S., Beier, C., and Michelsen, A. (2014). Soil microorganisms respond to five years of climate change manipulations and elevated atmospheric CO 2 in a temperate heath ecosystem. Plant Soil **374**:211–222. https://doi.org/10.1007/s11104-013-1855-1.

He, H., Wang, H., Fang, C., Wu, H., Guo, X., Liu, C., Lin, Z., and Lin, W. (2012). Barnyard grass stress up regulates the biosynthesis of phenolic compounds in allelopathic rice. J. Plant Physiol. **169**:1747–1753. https://doi.org/10.1016/j.jplph.2012.06.018.

Hernández Medina, R., Kutuzova, S., Nielsen, K.N., Johansen, J., Hansen, L.H., Nielsen, M., and Rasmussen, S. (2022). Machine learning and deep learning applications in microbiome research. ISME Commun. **2**:98. https://doi.org/10.1038/s43705-022-00182-9.

Herold, M., Martínez Arbas, S., Narayanasamy, S., Sheik, A.R., Kleine-Borgmann, L.A.K., Lebrun, L.A., Kunath, B.J., Roume, H., Bessarab, I., Williams, R.B.H., et al. (2020). Integration of time-series meta-omics data reveals how microbial ecosystems respond to disturbance. Nat. Commun. **11**:5281. https://doi.org/10.1038/s41467-020-19006-2.

Ho, T.K. (1995). Random decision forests. In Proceedings of 3rd international conference on document analysis and recognition (IEEE).

Horvath, R.S. (1972). Microbial co-metabolism and the degradation of organic compounds in nature. Bacteriol. Rev. **36**:146–155. https://doi.org/10.1128/br.36.2.146-155.1972.

Hou, D., Wang, R., Gao, X., Wang, K., Lin, Z., Ge, J., Liu, T., Wei, S., Chen, W., Xie, R., et al. (2018). Cultivar-specific response of bacterial community to cadmium contamination in the rhizosphere of rice (Oryza sativa L.). Environ. Pollut. **241**:63–73. https://doi.org/10.1016/j.envpol.2018.04.121.

Hunter, M.C., Smith, R.G., Schipanski, M.E., Atwood, L.W., and Mortensen, D.A. (2017). Agriculture in 2050: recalibrating targets for sustainable intensification. Bioscience **67**:386–391. https://doi.org/10.1093/biosci/bix010.

Ilsan, N.A., Nawangsih, A.A., and Wahyudi, A.T. (2015). Rice phyllosphere actinomycetes as biocontrol agent of bacterial leaf blight disease on rice. Asian J. Plant Pathol. **10**:1–8. https://doi.org/10.3923/ajppaj.2016.1.8.

Isawa, T., Yasuda, M., Awazaki, H., Minamisawa, K., Shinozaki, S., and Nakashita, H. (2010). Azospirillum sp. strain B510 enhances rice growth and yield. Microbes Environ. **25**:58–61. https://doi.org/10.1264/jsme2.me09174.

Jäderlund, L., Hellman, M., Sundh, I., Bailey, M.J., and Jansson, J.K. (2008). Use of a novel nonantibiotic triple marker gene cassette to monitor high survival of Pseudomonas fluorescens SBW25 on winter wheat in the field. FEMS Microbiol. Ecol. **63**:156–168. https://doi.org/10.1111/j.1574-6941.2007.00420.x.

Jain, D., Jones, L., and Roy, S. (2023). Gene editing to improve legume-rhizobia symbiosis in a changing climate. Curr. Opin. Plant Biol. **71**:102324. https://doi.org/10.1016/j.pbi.2022.102324.

Janßen, R., Beck, A.J., Werner, J., Dellwig, O., Alneberg, J., Kreikemeyer, B., Maser, E., Böttcher, C., Achterberg, E.P., Andersson, A.F., et al. (2021). Machine learning predicts the presence of 2, 4, 6-trinitrotoluene in sediments of a baltic sea munitions dumpsite using microbial community compositions. Front. Microbiol. **12**:626048. https://doi.org/10.3389/fmicb.2021.626048.

Jiang, X., Li, X., Yang, L., Liu, C., Wang, Q., Chi, W., and Zhu, H. (2019). How microbes shape their communities? A microbial community model based on functional genes. Dev. Reprod. Biol. **17**:91–105. https://doi.org/10.1016/j.gpb.2018.09.003.

Johansen, J., Plichta, D.R., Nissen, J.N., Jespersen, M.L., Shah, S.A., Deng, L., Stokholm, J., Bisgaard, H., Nielsen, D.S., Sørensen, S.J., et al. (2022). Genome binning of viral entities from bulk metagenomics data. Nat. Commun. **13**:965. https://doi.org/10.1101/2021.07.07.451412.

Jones, D.L., and Darrah, P.R. (1994). Role of root derived organic acids in the mobilization of nutrients from the rhizosphere. Plant Soil **166**:247–257. https://doi.org/10.1007/BF00008338.

Kaiser, R. (2006). Flowers and fungi use scents to mimic each other. Science **311**:806–807. https://doi.org/10.1126/science.1119499.

Karst, J., Gaster, J., Wiley, E., and Landhäusser, S.M. (2017). Stress differentially causes roots of tree seedlings to exude carbon. Tree Physiol. **37**:154–164. https://doi.org/10.1093/treephys/tpw090.

Kaster, A.-K., and Sobol, M.S. (2020). Microbial single-cell omics: the crux of the matter. Appl. Microbiol. Biotechnol. **104**:8209–8220. https://doi.org/10.1007/s00253-020-10844-0.

Kato-Noguchi, H. (2004). Allelopathic substance in rice root exudates: Rediscovery of momilactone B as an allelochemical. J. Plant Physiol. **161**:271–276. https://doi.org/10.1078/0176-1617-01188.

Kato-Noguchi, H., and Ino, T. (2005). Concentration and release level of momilactone B in the seedlings of eight rice cultivars. J. Plant Physiol. **162**:965–969. https://doi.org/10.1016/j.jplph.2005.02.007.

Kato, H., Kodama, O., and Akatsuka, T. (1993). Oryzalexin E, a diterpene phytoalexin from UV-irradiated rice leaves. Phytochemistry **33**:79–81. https://doi.org/10.1016/0031-9422(93)85399-C.

Ke, J., Wang, B., and Yoshikuni, Y. (2021). Microbiome engineering: synthetic biology of plant-associated microbiomes in sustainable agriculture. Trends Biotechnol. **39**:244–261. https://doi.org/10.1016/j.tibtech.2020.07.008.

Kim, K.-U., and Shin, D.-H. (2008). Progress and prospect of rice allelopathy research. In Allelopathy in Sustainable Agriculture and Forestry (Springer), pp. 189–213. https://doi.org/10.1007/978-0-387-77337-7.

Kim, S.Y., Lee, C.H., Gutierrez, J., and Kim, P.J. (2013). Contribution of winter cover crop amendments on global warming potential in rice paddy soil during cultivation. Plant Soil **366**:273–286. https://doi.org/10.1007/s11104-012-1403-4.

Kim, Y., and Oh, S. (2021). Machine-learning insights into nitrate-reducing communities in a full-scale municipal wastewater treatment plant. J. Environ. Manage. **300**:113795. https://doi.org/10.1016/j.jenvman.2021.113795.

Kodama, O., Miyakawa, J., Akatsuka, T., and Kiyosawa, S. (1992). Sakuranetin, a flavanone phytoalexin from ultraviolet-irradiated rice leaves. Phytochemistry **31**:3807–3809. https://doi.org/10.1016/S0031-9422(00)97532-0.

Koga, J., Shimura, M., Oshima, K., Ogawa, N., Yamauchi, T., and Ogasawara, N. (1995). Phytocassanes A, B, C and D, novel diterpene phytoalexins from rice, Oryza sativa L. Tetrahedron **51**:7907–7918. https://doi.org/10.1016/0040-4020(95)00423-6.

Kostic, A.D., Gevers, D., Siljander, H., Vatanen, T., Hyötyläinen, T., Hämäläinen, A.-M., Peet, A., Tillmann, V., Pöhö, P., Mattila, I., et al. (2015). The dynamics of the human infant gut microbiome in development and in progression toward type 1 diabetes. Cell Host Microbe **17**:260–273. https://doi.org/10.1016/j.chom.2015.01.001.

Krishanti, N., Wahyudi, A., and Nawangsih, A.A. (2015). Non-pathogenic phyllosphere bacteria producing bioactive compounds as biological control of Xanthomonas oryzae pv oryzae. Int. J. Pharma Bio Sci. **6**.

Kuang, X., Gu, J.-D., Tie, B., Yao, B., and Shao, J. (2016). Interactive effects of cadmium and Microcystis aeruginosa (cyanobacterium) on the growth, antioxidative responses and accumulation of cadmium and microcystins in rice seedlings. Ecotoxicology **25**:1588–1599. https://doi.org/10.1007/s10646-016-1714-y.

Kumar, M., Lee, S.-C., Kim, J.-Y., Kim, S.-J., Aye, S.S., and Kim, S.-R. (2014). Over-expression of dehydrin gene, OsDhn1, improves drought and salt stress tolerance through scavenging of reactive oxygen species in rice (Oryza sativa L.). J. Plant Biol. **57**:383–393. https://doi.org/10.1007/s12374-014-0487-1.

Lakshmanan, V., Cottone, J., and Bais, H.P. (2016). Killing two birds with one stone: natural rice rhizospheric microbes reduce arsenic uptake and blast infections in rice. Front. Plant Sci. **7**:1514. https://doi.org/10.3389/fpls.2016.01514.

Langille, M.G.I., Zaneveld, J., Caporaso, J.G., McDonald, D., Knights, D., Reyes, J.A., Clemente, J.C., Burkepile, D.E., Vega Thurber, R.L., Knight, R., et al. (2013). Predictive functional profiling of microbial communities using 16S rRNA marker gene sequences. Nat. Biotechnol. **31**:814–821. https://doi.org/10.1038/nbt.2676.

Layton, K.K.S., and Bradbury, I.R. (2022). Harnessing the power of multi-omics data for predicting climate change response. J. Anim. Ecol. **91**:1064–1072. https://doi.org/10.1111/1365-2656.13619.

Leach, J.E., Triplett, L.R., Argueso, C.T., and Trivedi, P. (2017). Communication in the phytobiome. Cell **169**:587–596. https://doi.org/10.1016/j.cell.2017.04.025.

Lebeis, S.L., Paredes, S.H., Lundberg, D.S., Breakfield, N., Gehring, J., McDonald, M., Malfatti, S., Glavina del Rio, T., Jones, C.D., Tringe, S.G., et al. (2015). Salicylic acid modulates colonization of the root microbiome by specific bacterial taxa. Science **349**:860–864. https://doi.org/10.1126/science.aaa8764.

Lee, J.-H., Wood, T.K., and Lee, J. (2015). Roles of indole as an interspecies and interkingdom signaling molecule. Trends Microbiol. **23**:707–718. https://doi.org/10.1016/j.tim.2015.08.001.

Levy, A., Salas Gonzalez, I., Mittelviefhaus, M., et al. (2018). Genomic features of bacterial adaptation to plants. Nat. Genet. **50**:138–150. https://doi.org/10.1038/s41588-017-0012-9.

Lewis, W.H., Tahon, G., Geesink, P., Sousa, D.Z., and Ettema, T.J.G. (2021). Innovations to culturing the uncultured microbial majority. Nat. Rev. Microbiol. **19**:225–240. https://doi.org/10.1038/s41579-020-00458-8.

Li, K., Wang, C., and Ow, D.W. (2022a). Root microbiome changes associated with cadmium exposure and/or overexpression of a transgene that reduces Cd content in rice. Ecotoxicol. Environ. Saf. **237**:113530. https://doi.org/10.1016/j.ecoenv.2022.113530.

Li, M., Xu, M., Su, A., Zhang, Y., Niu, L., and Xu, Y. (2022b). Combined phenanthrene and copper pollution imposed a selective pressure on the rice root-associated microbiome. Front. Microbiol. **13**:888086. https://doi.org/10.3389/fmicb.2022.888086.

Li, Y., Jian, X., Li, Y., Zeng, X., Xu, L., Khan, M.U., and Lin, W. (2020). OsPAL2-1 mediates allelopathic interactions between rice and specific microorganisms in the rhizosphere ecosystem. Front. Microbiol. **11**:1411. https://doi.org/10.3389/fmicb.2020.01411.

Lin, W.X. (2013). Rhizobiological properties of allelopathic rice in suppression of weeds and its research prospect. Acta Agron. Sin. **39**:951–960. https://doi.org/10.3724/SP.J.1006.2013.00951.

Liu, G.C., Tokida, T., Matsunami, T., Nakamura, H., Okada, M., Sameshima, R., Hasegawa, T., and Sugiyama, S.i. (2012). Microbial community composition controls the effects of climate change on methane emission from rice paddies. Environ. Microbiol. Rep. **4**:648–654. https://doi.org/10.1111/j.1758-2229.2012.00391.x.

Liu, H., and Brettell, L.E. (2019). Plant defense by VOC-induced microbial priming. Trends Plant Sci. **24**:187–189. https://doi.org/10.1016/j.tplants.2019.01.008.

Liu, K., and Bellet, A. (2019). Escaping the curse of dimensionality in similarity learning: Efficient Frank-Wolfe algorithm and generalization bounds. Neurocomputing **333**:185–199. https://doi.org/10.48550/arXiv.1807.07789.

Liu, Y.-X., Qin, Y., Chen, T., Lu, M., Qian, X., Guo, X., and Bai, Y. (2021). A practical guide to amplicon and metagenomic analysis of microbiome data. Protein Cell **12**:315–330. https://doi.org/10.1007/s13238-020-00724-8.

Liu, Y., Liu, X., Cheng, K., Li, L., Zhang, X., Zheng, J., Zheng, J., and Pan, G. (2016). Responses of methanogenic and methanotrophic communities to elevated atmospheric CO2 and temperature in a paddy field. Front. Microbiol. **7**:1895. https://doi.org/10.3389/fmicb.2016.01895.

Lo, C., and Marculescu, R. (2018). MetaNN: accurate classification of host phenotypes from metagenomic data using neural networks. In Proceedings of the 2018 ACM International Conference on Bioinformatics, Computational Biology, and Health Informatics. https://doi.org/10.1186/s12859-019-2833-2.

Lu, Y., Fu, L., Lu, Y., Hugenholtz, F., and Ma, K. (2015). Effect of temperature on the structure and activity of a methanogenic archaeal community during rice straw decomposition. Soil Biol. Biochem. **81**:17–27. https://doi.org/10.1016/j.soilbio.2014.10.031.

Lv, T., Zhan, C., Pan, Q., Xu, H., Fang, H., Wang, M., and Matsumoto, H. (2023). Plant pathogenesis: Toward multidimensional understanding of the microbiome. iMeta **2**:e129. https://doi.org/10.1002/imt2.129.

Lyu, D., and Smith, D.L. (2022). The root signals in rhizospheric inter-organismal communications. Front. Plant Sci. **13**:1064058. https://doi.org/10.3389/fpls.2022.1064058.

Madhaiyan, M., Poonguzhali, S., and Sa, T. (2007). Characterization of 1-aminocyclopropane-1-carboxylate (ACC) deaminase containing Methylobacterium oryzae and interactions with auxins and ACC

regulation of ethylene in canola (Brassica campestris). Planta **226**:867–876. https://doi.org/10.1007/s00425-007-0532-0.

Mano, H., and Morisaki, H. (2008). Endophytic bacteria in the rice plant. Microbes Environ. **23**:109–117. https://doi.org/10.1264/jsme2.23.109.

Marcos-Zambrano, L.J., Karaduzovic-Hadziabdic, K., Loncar Turukalo, T., Przymus, P., Trajkovik, V., Aasmets, O., Berland, M., Gruca, A., Hasic, J., Hron, K., et al. (2021). Applications of machine learning in human microbiome studies: a review on feature selection, biomarker identification, disease prediction and treatment. Front. Microbiol. **12**:634511. https://doi.org/10.3389/fmicb.2021.634511.

Mateu-Figueras, G., Pawlowsky-Glahn, V., and Egozcue, J.J. (2011). The principle of working on coordinates. Compositional data analysis: Theory and applications, pp. 29–42. https://doi.org/10.1002/9781119976462.ch3.

Mattiacci, L., Dicke, M., and Posthumus, M.A. (1995). beta-Glucosidase: an elicitor of herbivore-induced plant odor that attracts host-searching parasitic wasps. Proc. Natl. Acad. Sci. USA **92**:2036–2040. https://doi.org/10.1073/pnas.92.6.2036.

Mendes, R., Kruijt, M., De Bruijn, I., Dekkers, E., Van Der Voort, M., Schneider, J.H.M., Piceno, Y.M., DeSantis, T.Z., Andersen, G.L., Bakker, P.A.H.M., et al. (2011). Deciphering the rhizosphere microbiome for disease-suppressive bacteria. Science **332**:1097–1100. https://doi.org/10.1126/science.1203980.

Miles, P.W. (1999). Aphid saliva. Biological reviews **74**:41–85, 10.1073/pnas.92.6.2036. https://doi.org/10.1111/j.1469-185X.1999.tb00181.x.

Mitter, B., Pfaffenbichler, N., and Sessitsch, A. (2016). Plant–microbe partnerships in 2020. Microb. Biotechnol. **9**:635–640. https://doi.org/10.1111/1751-7915.12382.

Mizutani, J. (1999). Selected allelochemicals. Crit. Rev. Plant Sci. **18**:653–671. https://doi.org/10.1080/07352689991309432.

Moitinho-Silva, L., Steinert, G., Nielsen, S., Hardoim, C.C.P., Wu, Y.-C., McCormack, G.P., López-Legentil, S., Marchant, R., Webster, N., Thomas, T., et al. (2017). Predicting the HMA-LMA status in marine sponges by machine learning. Front. Microbiol. **8**:752. https://doi.org/10.3389/fmicb.2017.00752.

Msimbira, L.A., and Smith, D.L. (2020). The roles of plant growth promoting microbes in enhancing plant tolerance to acidity and alkalinity stresses. Front. Sustain. Food Syst. **4**:106. https://doi.org/10.3389/fsufs.2020.00106.

Mueller, U.G., and Sachs, J.L. (2015). Engineering microbiomes to improve plant and animal health. Trends Microbiol. **23**:606–617. https://doi.org/10.1016/j.tim.2015.07.009.

Naeimi, S., Okhovvat, S.M., Javan-Nikkhah, M., Vágvölgyi, C., Khosravi, V., and Kredics, L. (2010). Biological control of Rhizoctonia solani AG1-1A, the causal agent of rice sheath blight with Trichoderma strains. Phytopathol. Mediterr. **49**:287–300. https://doi.org/10.1038/s41467-022-28034-z.

Nearing, J.T., Douglas, G.M., Hayes, M.G., MacDonald, J., Desai, D.K., Allward, N., Jones, C.M.A., Wright, R.J., Dhanani, A.S., Comeau, A.M., et al. (2022). Microbiome differential abundance methods produce different results across 38 datasets. Nat. Commun. **13**:342.

Ning, J., and Beiko, R.G. (2015). Phylogenetic approaches to microbial community classification. Microbiome **3**:47. https://doi.org/10.1186/s40168-015-0114-5.

Omae, N., and Tsuda, K. (2022). Plant-microbiota interactions in abiotic stress environments. Mol. Plant Microbe Interact. **35**:511–526. https://doi.org/10.1094/MPMI-11-21-0281-FI.

Oudah, M., and Henschel, A. (2018). Taxonomy-aware feature engineering for microbiome classification. BMC Bioinf. **19**:227–313. https://doi.org/10.1186/s12859-018-2205-3.

Palmer, B., Guppy, C., Nachimuthu, G., and Hulugalle, N. (2023). Changes in micronutrient concentrations under minimum tillage and cotton-based crop rotations in irrigated Vertisols. Soil Tillage Res. **228**:105626. https://doi.org/10.1016/j.still.2022.105626.

Pasolli, E., Truong, D.T., Malik, F., Waldron, L., and Segata, N. (2016). Machine learning meta-analysis of large metagenomic datasets: tools and biological insights. PLoS Comput. Biol. **12**:e1004977. https://doi.org/10.1371/journal.pcbi.1004977.

Peltoniemi, K., Laiho, R., Juottonen, H., Bodrossy, L., Kell, D.K., Minkkinen, K., Mäkiranta, P., Mehtätalo, L., Penttilä, T., Siljanen, H.M., et al. (2016). Responses of methanogenic and methanotrophic communities to warming in varying moisture regimes of two boreal fens. Soil Biol. Biochem. **97**:144–156. https://doi.org/10.1016/j.soilbio.2016.03.007.

Peng, H., Long, F., and Ding, C. (2005). Feature selection based on mutual information criteria of max-dependency, max-relevance, and min-redundancy. IEEE Trans. Pattern Anal. Mach. Intell. **27**:1226–1238. https://doi.org/10.1109/TPAMI.2005.159.

Peng, J., Lü, Z., Rui, J., and Lu, Y. (2008). Dynamics of the methanogenic archaeal community during plant residue decomposition in an anoxic rice field soil. Appl. Environ. Microbiol. **74**:2894–2901. https://doi.org/10.1128/AEM.00070-08.

Phour, M., and Sindhu, S.S. (2023). Soil salinity and climate change: microbiome-based strategies for mitigation of salt stress to sustainable agriculture. In Climate Change and Microbiome Dynamics: Carbon Cycle Feedbacks (Springer), pp. 191–243. https://doi.org/10.1007/978-3-031-21079-2_13.

Pieterse, C.M., and Van Loon, L.C. (1999). Salicylic acid-independent plant defence pathways. Trends Plant Sci. **4**:52–58. https://doi.org/10.1016/s1360-1385(98)01364-8.

Poonguzhall, P., Selvaraj, S., Madhaiyan, M., and Sa, T. (2007). Production of acyl-homoserine lactone quorum-sensing signals is wide-spread in gram-negative Methylobacterium. J. Microbiol. Biotechnol. **17**:226–233. https://doi.org/10.1094/MPMI.1998.11.11.1119.

Porras, A.M., Shi, Q., Zhou, H., Callahan, R., Montenegro-Bethancourt, G., Solomons, N., and Brito, I.L. (2021). Geographic differences in gut microbiota composition impact susceptibility to enteric infection. Cell Rep. **36**:109457. https://doi.org/10.1016/j.celrep.2021.109457.

Qiu, D., Xiao, J., Ding, X., Xiong, M., Cai, M., Cao, Y., Li, X., Xu, C., and Wang, S. (2007). OsWRKY13 mediates rice disease resistance by regulating defense-related genes in salicylate-and jasmonate-dependent signaling. Mol. Plant Microbe Interact. **20**:492–499. https://doi.org/10.1094/MPMI-20-5-0492.

Qu, X.H., and Wang, J. (2008). Effect of amendments with different phenolic acids on soil microbial biomass, activity, and community diversity. Appl. Soil Ecol. **39**:172–179. https://doi.org/10.1016/j.apsoil.2007.12.007.

Quinn, T.P., Erb, I., Richardson, M.F., and Crowley, T.M. (2018). Understanding sequencing data as compositions: an outlook and review. Bioinformatics **34**:2870–2878. https://doi.org/10.1093/bioinformatics/bty175.

Ravanbakhsh, M., Sasidharan, R., Voesenek, L.A.C.J., Kowalchuk, G.A., and Jousset, A. (2018). Microbial modulation of plant ethylene signaling: ecological and evolutionary consequences. Microbiome **6**:52. https://doi.org/10.1186/s40168-018-0436-1.

Raza, A., Razzaq, A., Mehmood, S.S., Zou, X., Zhang, X., Lv, Y., and Xu, J. (2019). Impact of climate change on crops adaptation and strategies to tackle its outcome: A review. Plants **8**:34. https://doi.org/10.3390/plants8020034.

Rimando, A.M., Olofsdotter, M., Dayan, F.E., and Duke, S.O. (2001). Searching for rice allelochemicals: An example of bioassay-guided isolation. Agron. J. **93**:16–20. https://doi.org/10.2134/agronj2001.93116x.

Rodrigues, R.R., Rodgers, N.C., Wu, X., and Williams, M.A. (2018). COREMIC: a web-tool to search for a niche associated CORE MICrobiome. PeerJ **6**:e4395. https://doi.org/10.7717/peerj.4395.

Roth, R., Hillmer, S., Funaya, C., Chiapello, M., Schumacher, K., Lo Presti, L., Kahmann, R., and Paszkowski, U. (2019). Arbuscular cell invasion coincides with extracellular vesicles and membrane tubules. Nat. Plants **5**:204–211. https://doi.org/10.1038/s41477-019-0365-4.

Ruíz-Sánchez, M., Armada, E., Muñoz, Y., García de Salamone, I.E., Aroca, R., Ruíz-Lozano, J.M., and Azcón, R. (2011). Azospirillum and arbuscular mycorrhizal colonization enhance rice growth and physiological traits under well-watered and drought conditions. J. Plant Physiol. **168**:1031–1037. https://doi.org/10.1016/j.jplph.2010.12.019.

Sahu, D., Priyadarshani, I., and Rath, B. (2012). Cyanobacteria–as potential biofertilizer. CIBTech Journal of Microbiology **1**:20–26.

Saijo, Y., and Loo, E.P.i. (2020). Plant immunity in signal integration between biotic and abiotic stress responses. New Phytol. **225**:87–104. https://doi.org/10.1111/nph.15989.

Santos-Medellin, C., Liechty, Z., Edwards, J., Nguyen, B., Huang, B., Weimer, B.C., and Sundaresan, V. (2021). Prolonged drought imparts lasting compositional changes to the rice root microbiome. Nat. Plants **7**:1065–1077. https://doi.org/10.1038/s41477-021-00967-1.

Santos-Medellín, C., Edwards, J., Liechty, Z., Nguyen, B., and Sundaresan, V. (2017). Drought stress results in a compartment-specific restructuring of the rice root-associated microbiomes. mBio **8**:007644–e817. https://doi.org/10.1128/mBio.00764-17.

Schandry, N., and Becker, C. (2020). Allelopathic plants: models for studying plant–interkingdom interactions. Trends Plant Sci. **25**:176–185. https://doi.org/10.1016/j.tplants.2019.11.004.

Schloss, P.D., and Handelsman, J. (2005). Introducing DOTUR, a computer program for defining operational taxonomic units and estimating species richness. Appl. Environ. Microbiol. **71**:1501–1506. https://doi.org/10.1128/AEM.71.3.1501-1506.2005.

Schoch, C.L., Seifert, K.A., Huhndorf, S., Robert, V., Spouge, J.L., Levesque, C.A., Chen, W., and Fungal Barcoding Consortium; Fungal Barcoding Consortium Author List, and Bolchacova, E. (2012). Nuclear ribosomal internal transcribed spacer (ITS) region as a universal DNA barcode marker for Fungi. Proc. Natl. Acad. Sci. USA **109**:6241–6246. https://doi.org/10.1073/pnas.1117018109.

Seal, A.N., Pratley, J.E., Haig, T., and An, M. (2004). Identification and quantitation of compounds in a series of allelopathic and non-allelopathic rice root exudates. J. Chem. Ecol. **30**:1647–1662. https://doi.org/10.1023/b:joec.0000042074.96036.14.

Sengupta, S., Ganguli, S., and Singh, P.K. (2017). Metagenome analysis of the root endophytic microbial community of Indian rice (O. sativa L.). Genom. Data **12**:41–43. https://doi.org/10.1016/j.gdata.2017.02.010.

Senthilkumar, M., Madhaiyan, M., Sundaram, S., and Kannaiyan, S. (2009). Intercellular colonization and growth promoting effects of Methylobacterium sp. with plant-growth regulators on rice (Oryza sativa L. Cv CO-43). Microbiol. Res. **164**:92–104. https://doi.org/10.1016/j.micres.2006.10.007.

Shelake, R.M., Pramanik, D., and Kim, J.-Y. (2019). Exploration of plant-microbe interactions for sustainable agriculture in CRISPR era. Microorganisms **7**:269. https://doi.org/10.3390/microorganisms7080269.

Simon, J.-C., Marchesi, J.R., Mougel, C., and Selosse, M.-A. (2019). Host-microbiota interactions: from holobiont theory to analysis. Microbiome **7**:5. https://doi.org/10.1186/s40168-019-0619-4.

Singh, B.K., Trivedi, P., Singh, S., Macdonald, C.A., and Verma, J.P. (2018). Emerging microbiome technologies for sustainable increase in farm productivity and environmental security. Microbiol. Aust. **39**:17–23. https://doi.org/10.1071/MA18006.

Sivasakthi, S., Usharani, G., and Saranraj, P. (2014). Biocontrol potentiality of plant growth promoting bacteria (PGPR)-Pseudomonas fluorescens and Bacillus subtilis: A review. Afr. J. Agric. Res. **9**:1265–1277. https://doi.org/10.5897/AJAR2013.7914.

Sohrabi, R., Paasch, B.C., Liber, J.A., and He, S.Y. (2023). Phyllosphere microbiome. Annu. Rev. Plant Biol. **74**:539–568. https://doi.org/10.1146/annurev-arplant-102820-032704.

Song, F., Han, X., Zhu, X., and Herbert, S.J. (2012). Response to water stress of soil enzymes and root exudates from drought and non-drought tolerant corn hybrids at different growth stages. Can. J. Soil Sci. **92**:501–507. https://doi.org/10.4141/cjss2010-057.

Spence, C.A., Lakshmanan, V., Donofrio, N., and Bais, H.P. (2015). Crucial roles of abscisic acid biogenesis in virulence of rice blast fungus Magnaporthe oryzae. Front. Plant Sci. **6**:1082. https://doi.org/10.3389/fpls.2015.01082.

Statnikov, A., Henaff, M., Narendra, V., Konganti, K., Li, Z., Yang, L., Pei, Z., Blaser, M.J., Aliferis, C.F., and Alekseyenko, A.V. (2013). A comprehensive evaluation of multicategory classification methods for microbiomic data. Microbiome **1**:11–12. https://doi.org/10.1186/2049-2618-1-11.

Sultana, R., Islam, S.M.N., and Sultana, T. (2023). Arsenic and other heavy metals resistant bacteria in rice ecosystem: Potential role in promoting plant growth and tolerance to heavy metal stress. Environmental Technology & Innovation **31**:103160. https://doi.org/10.1016/j.eti.2023.103160.

Sun, H., Guo, X., Xu, F., Wu, D., Zhang, X., Lou, M., Luo, F., Xu, G., and Zhang, Y. (2019). Overexpression of OsPIN2 regulates root growth and formation in response to phosphate deficiency in rice. Int. J. Mol. Sci. **20**:5144. https://doi.org/10.3390/ijms20205144.

Sun, J., Miller, J.B., Granqvist, E., Wiley-Kalil, A., Gobbato, E., Maillet, F., Cottaz, S., Samain, E., Venkateshwaran, M., Fort, S., et al. (2015). Activation of symbiosis signaling by arbuscular mycorrhizal fungi in legumes and rice. Plant Cell **27**:823–838. https://doi.org/10.1105/tpc.114.131326.

Tang, Y., Bao, X., Zhi, Y., Wu, Q., Guo, Y., Yin, X., Zeng, L., Li, J., Zhang, J., He, W., et al. (2019). Overexpression of a MYB family gene, OsMYB6, increases drought and salinity stress tolerance in transgenic rice. Front. Plant Sci. **10**:168. https://doi.org/10.3389/fpls.2019.00168.

Tani, A., Sahin, N., Fujitani, Y., Kato, A., Sato, K., and Kimbara, K. (2015). Methylobacterium species promoting rice and barley growth and interaction specificity revealed with whole-cell matrix-assisted laser desorption/ionization-time-of-flight mass spectrometry (MALDI-TOF/MS) analysis. PLoS One **10**:e0129509. https://doi.org/10.1371/journal.pone.0129509.

Tarca, A.L., Carey, V.J., Chen, X.-w., Romero, R., and Drăghici, S. (2007). Machine learning and its applications to biology. PLoS Comput. Biol. **3**:e116. https://doi.org/10.1371/journal.pcbi.0030116.

Thapa, S., Prasanna, R., Ramakrishnan, B., Sheoran, N., Kumar, A., Velmourougane, K., and Kumar, A. (2018). Interactive effects of Magnaporthe inoculation and nitrogen doses on the plant enzyme machinery and phyllosphere microbiome of resistant and susceptible rice cultivars. Arch. Microbiol. **200**:1287–1305. https://doi.org/10.1007/s00203-018-1540-0.

Toju, H., Peay, K.G., Yamamichi, M., Narisawa, K., Hiruma, K., Naito, K., Fukuda, S., Ushio, M., Nakaoka, S., Onoda, Y., et al. (2018). Core microbiomes for sustainable agroecosystems. Nat. Plants **4**:247–257. https://doi.org/10.1038/s41477-018-0139-4.

Trivedi, P., Leach, J.E., Tringe, S.G., Sa, T., and Singh, B.K. (2020). Plant–microbiome interactions: from community assembly to plant health. Nat. Rev. Microbiol. **18**:607–621. https://doi.org/10.1038/s41579-020-0412-1.

Stotz, U., Brotherton, D., and Inal, J. (2022). Communication is key: extracellular vesicles as mediators of infection and defence during host–microbe interactions in animals and plants. FEMS Microbiol. Rev. **46**:fuab044. https://doi.org/10.1093/femsre/fuab044.

Uchida, A., Jagendorf, A.T., Hibino, T., Takabe, T., and Takabe, T. (2002). Effects of hydrogen peroxide and nitric oxide on both salt and heat stress tolerance in rice. Plant Sci. **163**:515–523. https://doi.org/10.1016/S0168-9452(02)00159-0.

UN. (2017). World population projected to reach 9.8 billion in 2050, and 11.2 billion in 2100 (United Nations).

Vandenkoornhuyse, P., Quaiser, A., Duhamel, M., Le Van, A., and Dufresne, A. (2015). The importance of the microbiome of the plant holobiont. New Phytol. **206**:1196–1206. https://doi.org/10.1111/nph.13312.

Vayssières, A., Pěnčík, A., Felten, J., Kohler, A., Ljung, K., Martin, F., and Legué, V. (2015). Development of the poplar-Laccaria bicolor ectomycorrhiza modifies root auxin metabolism, signaling, and response. Plant Physiol. **169**:890–902. https://doi.org/10.1104/pp.114.255620.

Verma, J.P. (2018). Functional Importance of the Plant Microbiome: Implications for Agriculture, Forestry and Bioenergy: A Book Review (Elsevier).

Videira, S.S., De Araujo, J.L.S., Rodrigues, L.d.S., Baldani, V.L.D., and Baldani, J.I. (2009). Occurrence and diversity of nitrogen-fixing Sphingomonas bacteria associated with rice plants grown in Brazil. FEMS Microbiol. Lett. **293**:11–19. https://doi.org/10.1111/j.1574-6968.2008.01475.x.

Viswanath, G., Sekar, J., and Ramalingam, P.V. (2020). Detection of diverse N-Acyl homoserine lactone signalling molecules among bacteria associated with rice rhizosphere. Curr. Microbiol. **77**:3480–3491. https://doi.org/10.1007/s00284-020-02183-0.

Vogel, E., Donat, M.G., Alexander, L.V., Meinshausen, M., Ray, D.K., Karoly, D., Meinshausen, N., and Frieler, K. (2019). The effects of climate extremes on global agricultural yields. Environ. Res. Lett. **14**:054010. https://doi.org/10.1088/1748-9326/ab154b.

Vorholt, J.A., Vogel, C., Carlström, C.I., and Müller, D.B. (2017). Establishing causality: opportunities of synthetic communities for plant microbiome research. Cell Host Microbe **22**:142–155. https://doi.org/10.1016/j.chom.2017.07.004.

Wang, E., Liu, X., Si, Z., Li, X., Bi, J., Dong, W., Chen, M., Wang, S., Zhang, J., Song, A., et al. (2021). Volatile organic compounds from rice rhizosphere bacteria inhibit growth of the pathogen Rhizoctonia solani. Agriculture **11**:368. https://doi.org/10.3390/agriculture11040368.

Wang, G., Weng, L., Huang, Y., Ling, Y., Zhen, Z., Lin, Z., Hu, H., Li, C., Guo, J., Zhou, J.L., et al. (2022). Microbiome-metabolome analysis directed isolation of rhizobacteria capable of enhancing salt tolerance of Sea Rice 86. Sci. Total Environ. **843**:156817. https://doi.org/10.1016/j.scitotenv.2022.156817.

Wang, H., He, H., Ye, C., Lu, J., Chen, R., Liu, C., Guo, X., and Lin, W. (2010). Molecular physiological mechanism of increased weed suppression ability of allelopathic rice mediated by low phosphorus stress. Allelopathy J. **25**:239–248.

Wang, J., Song, Y., Ma, T., Raza, W., Li, J., Howland, J.G., Huang, Q., and Shen, Q. (2017a). Impacts of inorganic and organic fertilization treatments on bacterial and fungal communities in a paddy soil. Appl. Soil Ecol. **112**:42–50. https://doi.org/10.1016/j.apsoil.2017.01.005.

Wang, Y., Tyler, B.M., and Wang, Y. (2019). Defense and counterdefense during plant-pathogenic oomycete infection. Annu. Rev. Microbiol. **73**:667–696. https://doi.org/10.1146/annurev-micro-020518-120022.

Wang, Y., Hatt, J.K., Tsementzi, D., Rodriguez-R, L.M., Ruiz-Pérez, C.A., Weigand, M.R., Kizer, H., Maresca, G., Krishnan, R., Poretsky, R., et al. (2017b). Quantifying the importance of the rare biosphere for microbial community response to organic pollutants in a freshwater ecosystem. Appl. Environ. Microbiol. **83**:e03321-16–e03316. https://doi.org/10.1128/AEM.03321-16.

Wang, Z., and Song, Y. (2022). Toward understanding the genetic bases underlying plant-mediated "cry for help" to the microbiota. IMeta **1**:e8. https://doi.org/10.1002/imt2.8.

Wari, D., Kabir, M.A., Mujiono, K., Hojo, Y., Shinya, T., Tani, A., Nakatani, H., and Galis, I. (2019). Honeydew-associated microbes elicit defense responses against brown planthopper in rice. J. Exp. Bot. **70**:1683–1696. https://doi.org/10.1093/jxb/erz041.

Watanabe, T., Katayanagi, N., Agbisit, R., Llorca, L., Hosen, Y., and Asakawa, S. (2021). Influence of alternate wetting and drying water-saving irrigation practice on the dynamics of Gallionella-related iron-oxidizing bacterial community in paddy field soil. Soil Biol. Biochem. **152**:108064. https://doi.org/10.1016/j.soilbio.2020.108064.

Weisburg, W.G., Barns, S.M., Pelletier, D.A., and Lane, D.J. (1991). 16S ribosomal DNA amplification for phylogenetic study. J. Bacteriol. **173**:697–703. https://doi.org/10.1128/jb.173.2.697-703.1991.

Weiss, S., Xu, Z.Z., Peddada, S., Amir, A., Bittinger, K., Gonzalez, A., Lozupone, C., Zaneveld, J.R., Vázquez-Baeza, Y., Birmingham, A., et al. (2017). Normalization and microbial differential abundance strategies depend upon data characteristics. Microbiome **5**:27. https://doi.org/10.1186/s40168-017-0237-y.

Welbaum, G.E., Sturz, A.V., Dong, Z., and Nowak, J. (2004). Managing soil microorganisms to improve productivity of agro-ecosystems. Crit. Rev. Plant Sci. **23**:175–193. https://doi.org/10.1080/07352680490433295.

Whipps, J.M., Lewis, K., and Cooke, R. (1988). Mycoparasitism and Plant Disease Control (Manchester: Manchester University Press).

Wirbel, J., Zych, K., Essex, M., Karcher, N., Kartal, E., Salazar, G., Bork, P., Sunagawa, S., and Zeller, G. (2021). Microbiome meta-analysis and cross-disease comparison enabled by the SIAMCAT machine learning toolbox. Genome biology **22**:1–27. https://doi.org/10.1101/2020.02.06.931808.

Wu, Y.-S., and Yang, C.-Y. (2019). Ethylene-mediated signaling confers thermotolerance and regulates transcript levels of heat shock factors in rice seedlings under heat stress. Bot. Stud. **60**:23. https://doi.org/10.1186/s40529-019-0272-z.

Xie, C.-H., and Yokota, A. (2006). Sphingomonas azotifigens sp. nov., a nitrogen-fixing bacterium isolated from the roots of Oryza sativa. Int. J. Syst. Evol. Microbiol. **56**:889–893. https://doi.org/10.1099/ijs.0.64056-0.

Xu, X., Xie, Z., Yang, Z., Li, D., and Xu, X. (2020). A t-SNE based classification approach to compositional microbiome data. Front. Genet. **11**:620143. https://doi.org/10.3389/fgene.2020.620143.

Xu, Z., Malmer, D., Langille, M.G.I., Way, S.F., and Knight, R. (2014). Which is more important for classifying microbial communities: who's there or what they can do? The ISME journal **8**:2357–2359. https://doi.org/10.1038/ismej.2014.157.

Yamada, S., Kano, A., Tamaoki, D., Miyamoto, A., Shishido, H., Miyoshi, S., Taniguchi, S., Akimitsu, K., and Gomi, K. (2012). Involvement of OsJAZ8 in jasmonate-induced resistance to bacterial

blight in rice. Plant Cell Physiol. **53**:2060–2072. https://doi.org/10.1093/pcp/pcs145.

Yasuda, M., Dastogeer, K.M.G., Sarkodee-Addo, E., Tokiwa, C., Isawa, T., Shinozaki, S., and Okazaki, S. (2022). Impact of Azospirillum sp. B510 on the rhizosphere microbiome of rice under field conditions. Agronomy **12**:1367. https://doi.org/10.3390/agronomy12061367.

Yue, J., Shi, Y., Zheng, X., Huang, G., and Zhu, J. (2007). The influence of free-air CO2 enrichment on microorganisms of a paddy soil in the rice-growing season. Appl. Soil Ecol. **35**:154–162. https://doi.org/10.1016/j.apsoil.2006.05.005.

Zhan, C., Matsumoto, H., Liu, Y., and Wang, M. (2022). Pathways to engineering the phyllosphere microbiome for sustainable crop production. Nat. Food **3**:997–1004. https://doi.org/10.1038/s43016-022-00636-2.

Zhang, J., Lin, G., Zhao, Q., and Zeng, D.-H. (2022). High adaptability of Pinus sylvestris var. mongolica to drought-induced soil nutrient deficiency. Ecol. Process. **11**:39. https://doi.org/10.1186/s13717-022-00381-1.

Zhu, X., and Troy, T.J. (2018). Agriculturally relevant climate extremes and their trends in the world's major growing regions. Earth's Future **6**:656–672. https://doi.org/10.1002/2017EF000687.

Plant Communications, Volume *5*

Supplemental information

Exploring and exploiting the rice phytobiome to tackle climate change challenges

Seyed Mahdi Hosseiniyan Khatibi, Niña Gracel Dimaano, Esteban Veliz, Venkatesan Sundaresan, and Jauhar Ali

Exploring and exploiting the rice phytobiome to tackle climate change challenges

Seyed Mahdi Hosseiniyan Khatibi,[1†] Niña Gracel Dimaano,[1,2†] Esteban Veliz,[3] Venkatesan Sundaresan,[3,4] Jauhar Ali[1]*

Affiliations/institutions

[1]International Rice Research Institute, Los Baños, Laguna, Philippines

[2]College of Agriculture and Food Science, University of the Philippines Los Baños, Laguna, Philippines

[3]College of Biological Sciences, UC Davis

[4]College of Agricultural and Environmental Sciences, UC Davis

***Corresponding author:**

Dr. Jauhar Ali

International Rice Research Institute, Los Baños, Laguna, Philippines

Email: J.Ali@irri.org

† These authors contributed equally and should be considered as co-first authors.

Short Summary:

The rice phytobiome is composed of an intricate network of interdependent and interrelating communities of micro- and macroorganisms and their surrounding environment. Dynamic interactions, facilitated by various communication signals, occur within the rice phytobiome that directly or indirectly influence rice plant growth, development, stress responses to environmental challenges, and overall rice ecosystem functioning. By using novel, data-driven, and systems-level approaches, the rice phytobiome can be accurately reprogrammed to combat climate change threats through the development of prescriptive and predictive analytics for the next-generation precision rice based agricultural systems.

This file includes:

Supplementary Table 1, 2

Supplementary Figure 1

References

Supplementary Table 1. Components of the rice phytobiome.

Component	Composition	Examples	Key roles/functions	References
Phyllosphere (aboveground) microbiomes				
a. Seed microbiome (outer grain, grain, husk, and outer husk)	Bacteria	*Sphingomonas*, *Acinetobacter*, *Aureimonas, Curtobacterium*, *Enterobacter*, *Exiguobacterium*, *Lactobacillus*, *Microbacterium*, *Pantoea*, *Pseudomonas*, *Methylobacterium fujisawaense*, *M. radiotolerans*, *Bacillus fusiformis*, *Kocuria palustris*, *Pantoea ananatis*, and *Micrococcus luteus,*	Enhance seed quality<br><br>Safeguard young seedlings from diseases<br><br>Increase plant fitness	(Nelson, 2018; Shade et al., 2017)
	Fungi	*Curvularia*, *Moesziomyces*, *Sacrocladium*, *Bipolaris*, *Fusarium equiseti*, *F. oxysporum*, *Epicoccum purpurascens*, *Cladosporium tenuissimum*, *Alternaria alternata*, and *Phoma sorghina*	Endophytic isolates of plant diseases or saprobes	(Fisher and Petrini, 1992)
b. Endosphere microbiome (inside spaces of a plant's stems, roots, leaves, fruits, and seeds)	Beneficial endophytes	*Microbacterium laevaniformans*, *Bacillus tequilensis, Micrococcus* sp., *Pseudomonas*, *Pseudomonas* sp., *Acremonium* sp., *Arthrobotrys* sp., *Fusarium* sp., *Penicillium* sp., *Aspergillus* sp., *Pyricularia* sp.	Positively modulate plant growth, productivity, and stress tolerance<br><br>Secrete secondary metabolites for protection against pathogen attack<br><br>Solubilize phosphate, produce siderophores, reduce sulfate and	(Hardoim et al., 2015; Jana et al., 2022)

			cyanide, and oxidize ammonia<br><br>Enhance water use efficiency<br><br>Induce systemic resistance in plants and stimulate plant growth	
	Commensal endophytes	*Penicillium* sp., *Aspergillus* sp.	Have neutral effects on plant performance	(Hardoim *et al.*, 2015; Rodriguez and Redman, 2008)
c. Plant microbiome (aboveground parts, including leaves, stems, and panicles)	Bacteria	*Proteobacteria*, *Firmicutes*, *Actinobacteria*, *Bacteroidetes*, *Cyanobacteria*, *Acinetobacter*, *Aureimonas*, *Curtobacterium*, *Enterobacter*, *Exiguobacterium*, *Lactobacillus*, *Microbacterium*, *Pantoea*, *Pseudomonas*, *Sphingomonas*	Beneficial, causing positive effects on overall plant growth of rice, or harmful, causing diseases of rice	(Peng et al., 2020)
	Fungi	Ascomycota (Eurotiomycetes, Dothideomycetes, and Sordariomycetes); Basidiomycota (Ustilaginomycetes); Aspergillus		(Peng *et al.*, 2020)
	Viruses	Rice Tungro Bacilliform Virus (RTBV) and Rice Stripe Virus (RSV)	Can cause viral diseases that have detrimental effects on rice productivity	(Deng et al., 2013; Hull, 1996)
	Bacteriophages	*Siphoviridae, Myoviridae*, and *Podoviridae*	Viruses that infect bacteria contribute to shaping the bacterial community structure within the rice phytobiome	(Jain et al., 2023; Zhang et al., 2021)

Rhizosphere and soil (belowground) microbiomes				
a. Rhizosphere microbiome (soil region surrounding the rice roots)	Bacteria	Acidobacteria, Chloroflexi, Firmicutes, and Proteobacteria	Involved in nutrient cycling, plant growth promotion, and disease suppression<br><br>Primarily influenced by factors relating to soil and plants, such as soil type, geographical location, and rice genotype, among others	(Ding et al., 2019; Edwards et al., 2015; Hussain et al., 2018; Santos et al., 2021)
	Fungi	Claroideoglomus, Gigaspora, Redeckera, Retroconis, Schizangiella, Ascomycota, Basidiomycota, and Arbuscular mycorrhiza	Enhance biotic resistance to soil-borne fungal and bacterial pathogens, nematodes, or root-chewing insects<br><br>Improve plant tolerance to abiotic stresses such as shading, high salinity, drought, and heavy metal contamination	(Chang et al., 2021; Wang et al., 2021; Xie et al., 2022)
	Archaea	Methanosaeta, Methanosarcina, Methanobacterium, Methanocella, and Methanoregula	Have a function in methane production	(Conrad et al., 2006; Wang et al., 2018)
	Nematode	Criconemella, Helicotylenchus, Hemicriconemoides, Hirschmanniella, Meloidogyne, and Tylenchorhynchus	Rice root parasites that can cause damage by disrupting the plant's vascular system, leading to decreased nutrient and water uptake<br><br>Predators of other soil-dwelling pests,	(Prot et al., 1994)

			including harmful nematodes and insect larvae<br><br>Certain nematodes have a symbiotic relationship with nitrogen-fixing bacteria	
b.Soil microbiome (soil surrounding a rice plant)	Bacteria	Chloroflexi, Proteobacteria, Acidobacteria, and Actinobacteria	Directly influence plant health and ecosystem functioning<br><br>Crucial for organic matter decomposition, nutrient cycling, and disease suppression	(Hussain et al., 2011; Jiang et al., 2016)
	Fungi	Glomeromycota, Basidiomycota, and Ascomycota		(Jiang et al., 2016; Yuan et al., 2018)
Macrobiome (macroorganims)				
Arthropods	Harmful insect pests	*Chilo suppressalis* (rice stem borer); *Nilaparvata lugens* (brown planthopper); *Nephotettix malayanus* and *N. virescens (green leafhoppers); Leptocorisa oratorius (rice seed bug); Scotinophara spp. (rice black bug); Spodoptera litura (common cutworm); Cnaphalocrocis medinalis (rice leaf folder)*	Damaging insect pests that attack plant parts, thus causing substantial yield losses	(Peng et al., 2016)
	Beneficial insects	*Cheilomenes sexmaculata* (ladybird beetles), *Chrysopa* sp. (lacewing), *Euborellia annulata* (earwigs), *Mantis religiosa* (European mantis), *Diadegma insulare* (parasitic wasp)	Benefit rice by controlling insect pest populations<br><br>Have ecological importance in maintaining rice ecosystem stability	(Peng et al., 2016; Ray and Chakraborty, 2021)
	Spiders	*Atypena formosana* (dwarf spider),	Prey on insect pests that damage the rice	(Ray and

		Oxyopes javanus (lynx spider), *Tetragnatha virescens* and *T. javana, Argiope catenulate*	plant, thus directly affecting the insect pest population	Chakraborty, 2021)
Mollusks and vertebrates	Snails and mollusks	*Pomacea canaliculata* and *P. maculata (golden apple snails)*	Highly invasive and cause damage to rice	(Horgan et al., 2014)
	Rats and birds	*Passer montanus* (Eurasian tree sparrow), *Lonchura malacca* (chesnut munia), *L. punctulata* (scaly-breasted munia), *L. leucogastra* (white-bellied munia)	Can cause yield damage to rice<br><br>Some birds can be beneficial by feeding on rice pests, thus diminishing their population	(Cohen et al., 1998)
Weeds	Grasses, broadleaves, and sedges	*Echinochloa* spp. (barnyard grass); *Cyperus difformis* (small flower umbrella plant); *Cyperus iria* (rice flatsedge); *Leptochloa chinensis* (sprangle top); *Ischaemum rugosum* (Saromacca grass); *Paspalum distichum* (knotgrass); *Fimbristylis littoralis* (fimbristylis); *Ludwigia octovalvis* (water purselane); *Pontederia vaginalis* (pickerel weed); *Sphenoclea zeylanica* (gooseweed)	Affect rice plants via interference through competition with limited resources or growth inhibition due to allelopathy<br><br>Can outcompete rice plants for soil nutrients and water, thus hampering their growth and development<br><br>Can compete for light, leading to shading and reduced photosynthetic activity<br><br>Allelochemicals produced by weed species inhibit rice seed germination and seedling growth	(Rao et al., 2017)

			Various weed species can serve as alternate hosts of many insect pests and diseases	
Human influences				
Chemical inputs	Chemical pesticides and fertilizers	herbicides, insecticides, fungicides, bactericides, molluscicides, chemical nutrient inputs/fertilizers	Have significant influences on plant-microbe interactions, soil health, and rice ecosystem dynamics<br><br>Control the population of insect pests, weeds, and pathogens and alleviate abiotic stresses related to nutrient deficiencies, respectively<br><br>Can alter the diversity and composition of microbial communities, leading to a decrease in beneficial microorganisms such as plant growth-promoting rhizobacteria<br><br>Disruption of the balance between beneficial and harmful organisms, as well as the development of resistance in the target insect pest populations, leading to more significant problems such as pest resurgence and replacement<br>Pesticide residues in rice fields can	(Lo, 2010)

			persist in the soil, thus affecting soil microbial communities, enzymatic activities, and nutrient cycling processes, and can lead to soil contamination and degradation	

Supplementary Table 2. Tasks and predictions in research on the microbiome.

Type of task	Aim of prediction	Suggested approaches	Reference
Analysis of interaction	Interaction of metabolite-microbe	Embedding method	(Brunner et al., 2023)
Analysis of interaction	Co-occurrence patterns of microbe	Embedding method	(Ge et al., 2023)
Analysis of interaction	Plant–pathogen interactions	Regression analysis	(Emmenegger et al., 2023)
Classification of microbial features	Composition of microbiome	Autoencoder method	(Busato et al., 2023)
Classification of microbial features	Profile of metabolic	Autoencoder method	(Li et al., 2023)
Phenotyping task	Disease suppressive soils	CNN method (Convolutional neural networks)	(Khatibi and Ali, 2024; Zhang et al., 2022)
Phenotyping task	Soil microbiome and crop productivity	Random forests	(Chang et al., 2017; Edwards et al., 2018)
Phenotyping task	Cd accumulation features	SVM, RF, LR, KNN, ANN	(Cheng et al., 2023)

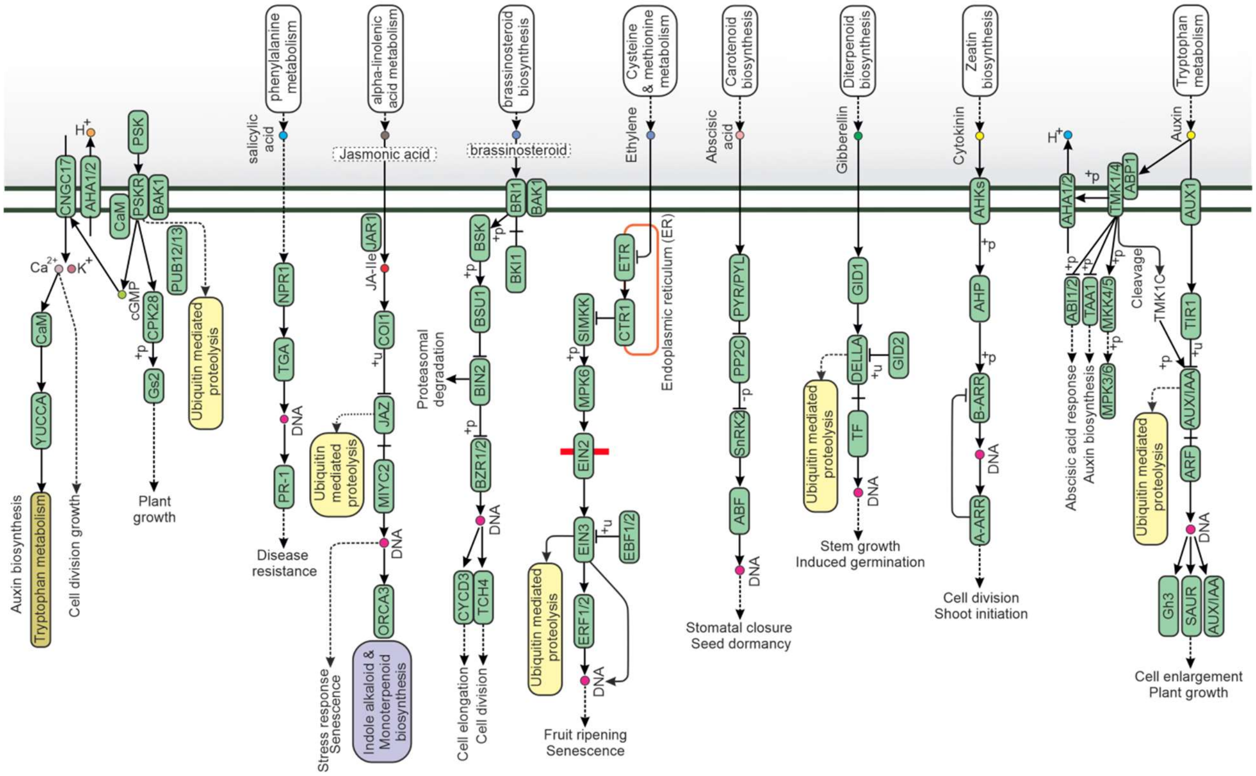

Supplementary Figure 1. The schematic illustration of the general hormones signaling pathways in the rice phytobiome

References

Brunner, J.D., Gallegos-Graves, L.A., and Kroeger, M.E. (2023). Inferring microbial interactions with their environment from genomic and metagenomic data. PLoS Computational Biology **19**:e1011661. https://doi.org/10.1371/journal.pcbi.1011661.

Busato, S., Gordon, M., Chaudhari, M., Jensen, I., Akyol, T., Andersen, S., and Williams, C. (2023). Compositionality, sparsity, spurious heterogeneity, and other data-driven challenges for machine learning algorithms within plant microbiome studies. Current Opinion in Plant Biology **71**:102326. https://doi.org/10.1016/j.pbi.2022.102326.

Chang, H.-X., Haudenshield, J.S., Bowen, C.R., and Hartman, G.L. (2017). Metagenome-wide association study and machine learning prediction of bulk soil microbiome and crop productivity. Frontiers in Microbiology **8**:519. https://doi.org/10.3389/fmicb.2017.00519.

Chang, J., Sun, Y., Tian, L., Ji, L., Luo, S., Nasir, F., Kuramae, E.E., and Tian, C. (2021). The structure of rhizosphere fungal communities of wild and domesticated rice: changes in diversity and co-occurrence patterns. Frontiers in Microbiology **12**:610823. https://doi.org/10.3389/fmicb.2021.610823.

Cheng, Z., Zheng, Q., Shi, J., He, Y., Yang, X., Huang, X., Wu, L., and Xu, J. (2023). Metagenomic and machine learning-aided identification of biomarkers driving distinctive Cd accumulation features in the root-associated microbiome of two rice cultivars. ISME communications **3**:14. https://doi.org/10.1038/s43705-023-00213-z

Cohen, M., Savary, S., Huang, N., Azzam, O., and Datta, S. (1998). Importance of rice pests and challenges to their management. See Ref **30**:145-164.

Conrad, R., Erkel, C., and Liesack, W. (2006). Rice Cluster I methanogens, an important group of Archaea producing greenhouse gas in soil. Current Opinion in Biotechnology **17**:262-267. https://doi.org/10.1016/j.copbio.2006.04.002.

Deng, X., Wang, H., Tang, X., Zhou, J., Chen, H., He, G., Chen, L., and Xu, Z. (2013). Hybrid rice breeding welcomes a new era of molecular crop design. Sci. Sin. Vitae **43**:864-868. https://doi.org/10.1360/052013-299.

Ding, L.-J., Cui, H.-L., Nie, S.-A., Long, X.-E., Duan, G.-L., and Zhu, Y.-G. (2019). Microbiomes inhabiting rice roots and rhizosphere. FEMS microbiology ecology **95**:fiz040. https://doi.org/10.1093/femsec/fiz040.

Edwards, J., Johnson, C., Santos-Medellín, C., Lurie, E., Podishetty, N.K., Bhatnagar, S., Eisen, J.A., and Sundaresan, V. (2015). Structure, variation, and assembly of the root-associated microbiomes of rice. Proceedings of the National Academy of Sciences **112**:E911-E920. https://doi.org/10.1073/pnas.1414592112.

Edwards, J.A., Santos-Medellín, C.M., Liechty, Z.S., Nguyen, B., Lurie, E., Eason, S., Phillips, G., and Sundaresan, V. (2018). Compositional shifts in root-associated bacterial and archaeal microbiota track the plant life cycle in field-grown rice. PLoS biology **16**:e2003862. https://doi.org/10.1371/journal.pbio.2003862.

Emmenegger, B., Massoni, J., Pestalozzi, C.M., Bortfeld-Miller, M., Maier, B.A., and Vorholt, J.A. (2023). Identifying microbiota community patterns important for plant protection using synthetic communities and machine learning. Nature Communications **14**:7983. https://doi.org/10.1038/s41467-023-43793-z.

Fisher, P., and Petrini, O. (1992). Fungal saprobes and pathogens as endophytes of rice (Oryza sativa L.). New Phytologist **120**:137-143. https://doi.org/10.1111/j.1469-8137.1992.tb01066.x.

Ge, J., Li, D., Ding, J., Xiao, X., and Liang, Y. (2023). Microbial coexistence in the rhizosphere and the promotion of plant stress resistance: a review. Environmental Research **222**:115298. https://doi.org/10.1016/j.envres.2023.115298.

Hardoim, P.R., Van Overbeek, L.S., Berg, G., Pirttilä, A.M., Compant, S., Campisano, A., Döring, M., and Sessitsch, A. (2015). The hidden world within plants: ecological and evolutionary considerations for defining functioning of microbial endophytes. Microbiology and molecular biology reviews **79**:293-320. https://doi.org/10.1128/MMBR.00050-14.

Horgan, F.G., Stuart, A.M., and Kudavidanage, E.P. (2014). Impact of invasive apple snails on the functioning and services of natural and managed wetlands. Acta Oecologica **54**:90-100. https://doi.org/10.1016/j.actao.2012.10.002.

Hull, R. (1996). Molecular biology of rice tungro viruses. Annual review of phytopathology **34**:275-297. https://doi.org/10.1146/annurev.phyto.34.1.275.

Hussain, M., Hamid, M.I., Tian, J., Hu, J., Zhang, X., Chen, J., Xiang, M., and Liu, X. (2018). Bacterial community assemblages in the rhizosphere soil, root endosphere and cyst of soybean cyst nematode-suppressive soil challenged with nematodes. FEMS microbiology ecology **94**:fiy142. https://doi.org/10.1093/femsec/fiy142.

Hussain, Q., Liu, Y., Zhang, A., Pan, G., Li, L., Zhang, X., Song, X., Cui, L., and Jin, Z. (2011). Variation of bacterial and fungal community structures in the rhizosphere of hybrid and standard rice cultivars and linkage to CO2 flux. FEMS Microbiology Ecology **78**:116-128. https://doi.org/10.1111/j.1574-6941.2011.01128.x.

Jain, L., Kumar, V., Jain, S.K., Kaushal, P., and Ghosh, P.K. (2023). Isolation of bacteriophages infecting Xanthomonas oryzae pv. oryzae and genomic characterization of novel phage vB_XooS_NR08 for biocontrol of bacterial leaf blight of rice. Frontiers in Microbiology **14**:1084025. https://doi.org/10.3389/fmicb.2023.1084025.

Jana, S.K., Islam, M.M., and Mandal, S. (2022). Endophytic microbiota of rice and their collective impact on host fitness. Current Microbiology **79**:37. https://doi.org/10.1007/s00284-021-02737-w.

Jiang, Y., Liang, Y., Li, C., Wang, F., Sui, Y., Suvannang, N., Zhou, J., and Sun, B. (2016). Crop rotations alter bacterial and fungal diversity in paddy soils across East Asia. Soil Biology and Biochemistry **95**:250-261. https://doi.org/10.1016/j.soilbio.2016.01.007.

Khatibi, S.M.H., and Ali, J. (2024). Harnessing the power of machine learning for crop improvement and sustainable production. Frontiers in Plant Science 15:1417912. https://doi.org/10.3389/fpls.2024.1417912.

Li, J., Gmitter Jr, F.G., Zhang, B., and Wang, Y. (2023). Uncovering Interactions between Plant Metabolism and Plant-Associated Bacteria in Huanglongbing-Affected Citrus Cultivars Using Multiomics Analysis and Machine Learning. Journal of Agricultural and Food Chemistry **71**:16391-16401. https://doi.org/10.1021/acs.jafc.3c04460.

Lo, C.-C. (2010). Effect of pesticides on soil microbial community. Journal of Environmental Science and Health Part B **45**:348-359. https://doi.org/10.1002/2688-8319.12327.

Nelson, E.B. (2018). The seed microbiome: origins, interactions, and impacts. Plant and Soil **422**:7-34. https://doi.org/10.1007/s11104-017-3289-7.

Peng, L., Zhao, Y., Wang, H., Zhang, J., Song, C., Shangguan, X., Zhu, L., and He, G. (2016). Comparative metabolomics of the interaction between rice and the brown planthopper. Metabolomics **12**:1-15. https://doi.org/10.1007/s11306-016-1077-7.

Peng, Y., Tang, J., Hong, M., and Xie, J. (2020). Suppression of rice planthopper populations by the entomopathogenic fungus Metarhizium anisopliae without affecting the rice microbiota. Applied and Environmental Microbiology **86**:e01337-01320. https://doi.org/10.1128/AEM.01337-20.

Prot, J.-C., Soriano, I., and Matias, D. (1994). Major root-parasitic nematodes associated with irrigated rice in the Philippines. Fundamental and Applied Nematology **17**:75-78.

Rao, A., Wani, S., Ahmed, S., Haider Ali, H., and Marambe, B. (2017). An overview of weeds and weed management in rice of South Asia.

Ray, A., and Chakraborty, A. (2021). The edible biota in irrigated, deepwater, and rainfed rice fields of Asia: a neglected treasure for sustainable food system. Environment, Development and Sustainability **23**:17163-17179. https://doi.org/10.1007/s10668-021-01386-0.

Rodriguez, R., and Redman, R. (2008). More than 400 million years of evolution and some plants still can't make it on their own: plant stress tolerance via fungal symbiosis. Journal of experimental botany **59**:1109-1114. https://doi.org/10.1093/jxb/erm342.

Santos, S.S., Rask, K.A., Vestergård, M., Johansen, J.L., Priemé, A., Frøslev, T.G., González, A.M.M., He, H., and Ekelund, F. (2021). Specialized microbiomes facilitate natural rhizosphere microbiome interactions counteracting high salinity stress in plants. Environmental and Experimental Botany **186**:104430. https://doi.org/10.1016/j.envexpbot.2021.104430.

Shade, A., Jacques, M.-A., and Barret, M. (2017). Ecological patterns of seed microbiome diversity, transmission, and assembly. Current opinion in microbiology **37**:15-22. https://doi.org/10.1016/j.mib.2017.03.010.

Wang, C., Jin, Y., Ji, C., Zhang, N., Song, M., Kong, D., Liu, S., Zhang, X., Liu, X., and Zou, J. (2018). An additive effect of elevated atmospheric CO2 and rising temperature on methane emissions related to methanogenic community in rice paddies. Agriculture, Ecosystems & Environment **257**:165-174. https://doi.org/10.1016/j.agee.2018.02.003.

Wang, Y., Bao, X., and Li, S. (2021). Effects of arbuscular mycorrhizal fungi on rice growth under different flooding and shading regimes. Frontiers in Microbiology **12**:756752. https://doi.org/10.3389/fmicb.2021.756752.

Xie, H., Chen, Z., Feng, X., Wang, M., Luo, Y., Wang, Y., and Xu, P. (2022). L-theanine exuded from Camellia sinensis roots regulates element cycling in soil by shaping the rhizosphere microbiome

assembly. Science of The Total Environment **837**:155801. https://doi.org/10.1016/j.scitotenv.2022.155801.

Yuan, J., Zhao, J., Wen, T., Zhao, M., Li, R., Goossens, P., Huang, Q., Bai, Y., Vivanco, J.M., and Kowalchuk, G.A. (2018). Root exudates drive the soil-borne legacy of aboveground pathogen infection. Microbiome **6**:1-12. https://doi.org/10.1186/s40168-018-0537-x.

Zhang, J., Cook, J., Nearing, J.T., Zhang, J., Raudonis, R., Glick, B.R., Langille, M.G., and Cheng, Z. (2021). Harnessing the plant microbiome to promote the growth of agricultural crops. Microbiological Research **245**:126690. https://doi.org/10.1016/j.micres.2020.126690.

Zhang, Z., Zhang, Q., Cui, H., Li, Y., Xu, N., Lu, T., Chen, J., Penuelas, J., Hu, B., and Qian, H. (2022). Composition identification and functional verification of bacterial community in disease-suppressive soils by machine learning. Environmental Microbiology **24**:3405-3419. https://doi.org/10.1111/1462-2920.15902.
